# Overcoming off-targets: assessing Western blot signals for Bcnt/Cfdp1, a tentative component of the chromatin remodeling complex

**DOI:** 10.1042/BSR20194012

**Published:** 2020-06-09

**Authors:** Shintaro Iwashita, Takehiro Suzuki, Yoshimitsu Kiriyama, Naoshi Dohmae, Yoshiharu Ohoka, Si-Young Song, Kentaro Nakashima

**Affiliations:** 1 Kagawa School of Pharmaceutical Sciences, Tokushima Bunri University, Shido 1314-1, Sanuki, Kagawa 769-2193, Japan; 2 Biomolecular Characterization Unit, RIKEN Center for Sustainable Resource Science, Saitama 351-0198, Japan

**Keywords:** antibody evaluation, endogenous Bcnt/Cfdp1, Mass spectroscopy, Phosphoprotein, RNA sequence analysis, Western blot analysis

## Abstract

The Bucentaur (BCNT) protein family is characterized by a conserved amino acid sequence at the C-terminus (BCNT-C domain) and plays an essential role in gene expression and chromosomal maintenance in yeast and *Drosophila*. The mammalian Bucentaur/Craniofacial developmental protein 1 (Bcnt/Cfdp1) is also a tentative component of the SNF2-related CBP activator protein (Srcap) chromatin remodeling complex, but little is known about its properties, partly because few antibodies are available to examine the endogenous protein. In this paper, we assigned the Western blot signal against the mouse Bcnt/Cfdp1 as a doublet of approximately 45 kDa using anti-Bcnt/Cfdp1 antibodies, which were generated against either of two unrelated immunogens, BCNT-C domain or mouse N-terminal peptide, and in addition, the Cfdp1 knockdown mouse ES cell line and bovine tissue were used as potential negative controls. Moreover, LC-MS/MS analysis of the corresponding doublet to the Flag-tagged mouse Bcnt/Cfdp1 that was constitutively expressed in a HEK293 cell exhibited that the upper band was much more phosphorylated than the lower band with preferential Ser phosphorylation in the WESF motif of BCNT-C domain. Western blot analysis with these evaluated antibodies indicated a preferential expression of Bcnt/Cfdp1 in the early stages of brain development of mouse and rat, which is consistent with a data file of the expression of Bcnt/Cfdp1 mRNA.

## Introduction

The BCNT family members in yeast and *Drosophila* have been shown to play essential roles in gene expression and chromosomal maintenance [1]. Mammalian Bcnt/Cfdp1 is also presumed to be a component of the SNF2-related CBP activator protein (Srcap) chromatin remodeling complex, which is based on the results using fractionation of cultured human cell extracts followed by mass spectrometry [2]. This presumed frame is originally based on the analysis of Swc5, a budding yeast ortholog of Bcnt/Cfdp1, in Swr1 (yeast Srcap) chromatin complex [3–5]. Although Swc5 is not integrated with the Swr1 complex, it participates in the activation of the remodeler ATPase, Srcap, and the ATP-dependent histone exchange reaction. The reaction replaces nucleosomal H2A–H2B with H2A.Z–H2B dimers by recruiting the variant H2A.Z in the transcription and DNA repair [6–8]. *Swc5* is not essential for survival, yet its deletion mutant *swc*5Δ cells caused lack in histone replacement activity for Swr1, resulting in genetic instability, hypersensitivity to drugs, and transcriptional misregulation [9], [The *Saccharomyces* Genome Database https://www.yeastgenome.org/].

**Figure 1:**
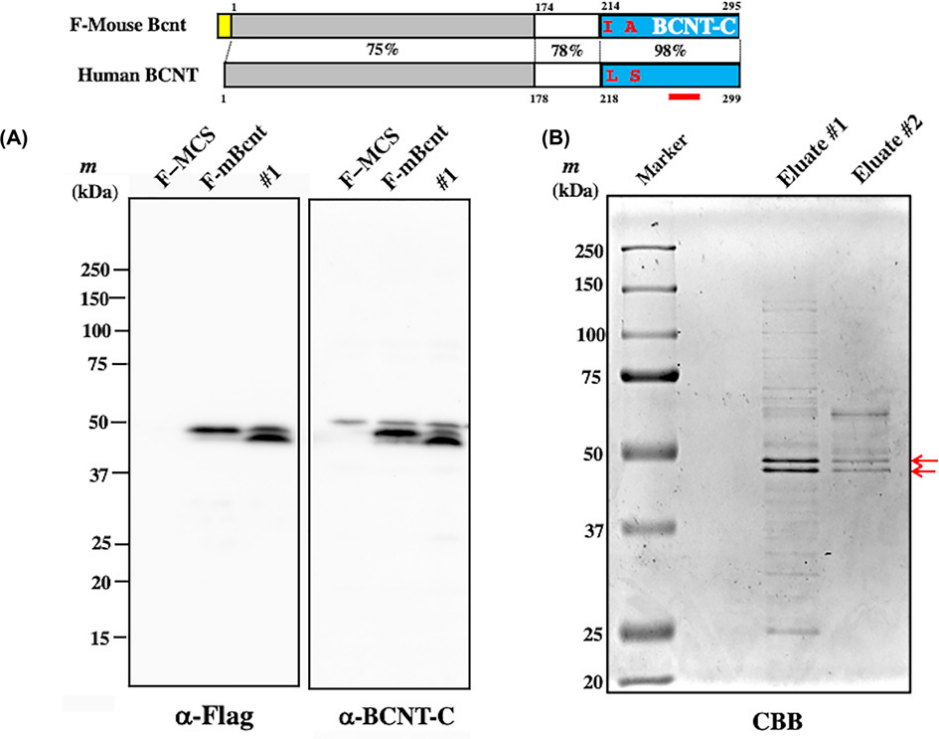
Domain structure of flag-tagged mBcnt, its expression as a doublet band, and isolation of each band **Top panel**: Structural comparison of human BCNT/CFDP1 (Human BCNT) and mouse Bcnt/Cfdp1 (Mouse Bcnt). mBcnt is composed of 295 amino acids and 4 amino acids less than the human counterpart. While the C-terminal 82 amino acid sequence of the BCNT-C domain (blue boxes) is identical except for two amino acid residues (red letters), the N-terminal region has low homology between mouse and human (75%). A yellow box attached to the N-terminal of mBcnt shows Flag-tag. (**A**) Flag-tagged mBcnt (F-mBcnt) was detected as a doublet in both transient and constitutive expression. F-mBcnt or F-multi-cloning site (MCS) was expressed in T-REx cells, and the extracts were prepared after culturing for 46 h as transiently expressed samples (Lanes: F-MCS and F-mBcnt). In addition, the extract of several candidate colonies that constitutively expressed F-mBcnt were prepared (Supplementary Figure S1) and their representative #1 (from ∼5 × 10^4^ cells) was presented. These extracts were subjected to a Western blot analysis with anti-Flag antibody (Ab) (left panel) or anti-BCNT-C Ab (right panel). The red bar below the structure of Bcnt indicates the location of immunogen for anti-BCNT-C Ab production. (**B**) Isolation of the upper and lower bands of F-mBcnt doublet from the #1 colony*.* The supernatant of the #1 colony extract isolated by centrifugation was mixed with anti-Flag-tag agarose beads, and the adsorbed fraction was isolated by sequential elution with Flag peptide (Eluate #1 and #2), as shown in Supplementary Figure S2. After evaluation of the chromatogram, the larger preparation was carried out on SDS/PAGE and detected by Coomassie Brilliant Blue staining (CBB). The arrows indicate a doublet band.

Members of the Bcnt family generally consist of an acidic N-terminal region, a highly conserved C-terminal region with approximately 80 amino acids (BCNT-C domain), and a hydrophilic region between them (Figure 1 and [1]). Recently, the BCNT-C domain of the budding yeast Swc5 was found to be essential for the histone exchange reaction [10]. On the other hand, a deletion mutant induced by one-base replacement of the *Drosophila* ortholog, *Yeti*, which entirely lacks the BCNT-C domain, shows substantial chromosomal abnormalities, resulting in lethality before pupation [11]. Thus, the metazoan *Bcnt/Cfdp1* is essential for survival in contrast to the yeast *swc5*. Furthermore, the chicken ortholog CENP-29 has been identified to be a kinetochore-associated protein [12]. Given a report that CENP-B protects centromere chromatin integrity by promoting histone deposition [13], these results imply that the Bcnt members may play broader roles in the maintenance of the structure and function of the chromosome.

RNA sequencing analysis, as shown by the dramatic influence caused by genetic mutations of *swc5* in fission yeast [14], is a powerful method to clarify the mechanism of complex and dynamic processes, such as embryonic development and stress adaptation. However, recent studies have shown a discordance between mRNA and protein expression in such dynamic processes and argue that analysis at the transcriptional level is insufficient to predict protein levels [15]. Since members of the Bcnt family may preferentially function to maintain the structure and function of the chromosome in these dynamic processes, analysis of their protein dynamics is essential to understand those processes. However, it is challenging to assign the correct signal by most of the currently available Bcnt/Cfdp1 antibodies (Abs), even in Western blot analysis.

We previously characterized human BCNT/CFDP1 (hBCNT/CFDP1) using a cell line that constitutively expresses its His-tagged molecule (His-hBCNT/CFDP1) [16] as well as Ab generated against an 18-mer peptide (EELAIHNRGKEGYIERKA) derived from BCNT-C (anti-BCNT-C Ab) (Table 1) [17]. His-hBCNT/CFDP1 has a calculated mass of 34.9 kDa (33.6 kDa plus His-tag), but its immunoreactive signal was detected around 50 kDa as a doublet band on SDS-polyacrylamide gel electrophoresis (SDS/PAGE). We showed that the difference between its calculated and apparent molecular mass is mainly due to the acidic stretch located in the N-terminal region and Ser^250^ phosphorylation in the BCNT-C domain [16]. However, we could not identify the endogenous hBCNT/CFDP1 due to a high background caused by anti-His Ab reactive proteins, and we also could not accurately assess the specificity of anti-BCNT-C Ab to endogenous hBCNT/CFDP1. Furthermore, we recently found that the anti-BCNT-C Ab cross-reacts with an unrelated target, glutamine synthetase (GS) (mouse GS, NP_032157; EC 6.3.1.2), which is also known as γ-glutamate: ammonia ligase [18]. In this paper, we assigned the Western blot signal against the endogenous mouse Bcnt/Cfdp1 (mBcnt/Cfdp1) using various target-related materials, including flag-tagged mBcnt/Cfdp1 (F-mBcnt/Cfdp1) and *Bcnt/Cfdp1* knockdown ES cells. We also present a scheme to prepare a potential negative control for the western blot analysis to evaluate the Bcnt/Cfdp1 signal. Then, we demonstrate a high expression of Bcnt/Cfdp1 at an early developmental stage of the brains of mice and rats by evaluating the Abs.

**Table 1:** Properties of four anti-Bcnt/Cfdp1 Abs used in the study

Antibody name	Immunized animal	Antigen (Sequences/Location)	Properties of antigens or Abs	Reference
**Anti-BCNT-C Ab**	Guinea pig	NH_2_-C**EELAIHNRGKEGYIERKA**-COOH Human BCNT/CFDP1 259-276^th^ peptide	Common to almost mammals	[17,18]
**Anti**-**mBcnt-N Ab**	Guinea pig	CH_3_CO-**GEEQAEKTKGKRRKAQ**C-COOH Mouse Bcnt/Cfdp1 41-56^th^ peptide	Mouse and rat specific	[18]
**A305-624A-M** (BethyI)	Rabbit	Human BCNT/CFDP1 249-299^th^ petide https://www.bethyl.com/product/a305-624A-M/CFDP1+Antibody)	Detection of ∼48, ∼37, and ∼19 kDa bands, immunoprecipitation ability	
**26636-1-AP** (Proteintech)	Rabbit	Human BCNT/CFDP1 172-299^th^ Recombinant https://www.ptglab.com/products/CFDP1-Antibody-26636-1-AP.htm	Validated by siRNA knockdown	

Amino acid residues of antigen peptides are indicated in bold letters and their cysteine residues for conjugation with keyhole limpet hemocyanin are also shown.

## Materials and methods

Detailed information on material sources are listed in Supplementary Table S4.

### Cell culture

T-REx-293 (a HEK293 cell derivative, T-REx) cells, their subcolonies which express Flag-multi-cloning site (F-MCS) or F-mBcnt, and their clone G11 cells which express His-hBCNT/CFDP1 [16] were routinely maintained in a 5% CO_2_ incubator using DMEM-GlutaMAX-1 (DMEM) supplemented with 10% fetal calf serum, 50 μg/ml gentamicin, and with or without G418 (0.5 mg/ml). These cells were subcultured by incubating cell layers (2 ml medium in a 35-mm dish) with 0.75 ml of Accutase for 5 min at room temperature, dispersed by pipetting with a 1000-μl pipet tip, and ∼1/15 of the suspension was directly plated on a dish preincubated with the medium. G418 was added on the next day when needed. For preparation of cellular protein extract or transfection, after washing the cell layers (5 ml of medium in a 60-mm dish or 10 ml of medium in a 100-mm dish) with prewarmed Hepes buffered saline (HBS [10 mM Hepes-NaOH, pH 7.5, 150 mM NaCl]), their cell suspension were prepared as described above with the medium. TrypLE Express was also used in the earlier stage of the study (see below).

### ES cell culture

35-mm dishes were precoated by incubating with recombinant human Laminin (iMatrix-511) at a final concentration of 5 μg/ml in phosphate-buffered saline (PBS) either at room temperature or at 37°C. Cfdp1-K1 (cell No: AyuK7D01) and vdR2-4 (cell No: JCRB1658) cells were obtained from Japanese Collection of Research Bioresources (JCRB), and were grown on precoated 35-mm dishes in ESGRO Complete Clonal Grade Medium plus GSK3β Inhibitor (50 μl/100 ml), further supplemented with gentamicin (50 μg/ml). After washing with prewarmed HBS, cell layers (2 ml medium in a 35-mm dish) were incubated with 0.75 ml of Accutase at room temperature and dispersed by pipetting with a 1000-μl pipet tip after 5 min, and 1 ml of DMEM containing 0.1% polyvinyl alcohol (DMEM-PVA) was added. The cells were collected into a 15-ml tube after washing the dish with DMEM-PVA. While counting its cell number, the suspension was centrifuged (100 x *g*, 3 min, room temperature), resuspended in a new medium and seeded at the density of ∼ 2.5 × 10^5^ cells per 35-mm dish. In order to prepare protein extracts or total RNA, cell suspensions were washed twice with chilled HBS, dispensed at ∼1 × 10^6^ cells per 1.5-ml tube, and centrifuged (100 × ***g***, 5 min, 4°C). After removing the buffer, cell pellets were softly vortexed, snap-frozen in liquid nitrogen, and stocked at −80°C until use.

### Generation of T-REx colonies expressing Flag-mBcnt

T-REx cells (∼2 × 10^6^ per 100-mm dish) were culture for one day, transfected with each 5 μg of Flag-MCS-pcDNA3.1 (Accession No. LC311018) or Flag-MCS-pcDNA3.1 plasmid carrying m*Bcn*t using Lipofectamine 3000 according to the manufacturer’s protocol. Just before transfection, each half medium was once removed, saved, and returned to the culture ∼5 h after transfection. After culturing for a total of 44–48 h, each cell layer was washed with prewarmed HBS, dispersed with TrypLE Express for 10 min at 37°C, harvested with the medium into a 15-ml tube, centrifuged, and resuspended in the medium. The number of resuspended cells was counted, and ∼60 cells were seeded in a 48-well plate preincubated with the medium. After 4 h, G418 was added to a final concentration of 0.75 mg/ml. Colonies growing in 48-well plates were sequentially expanded to 24-well plates and 35-mm dishes. Each colony was then cultured in the presence of 0.5 mg/ml G418.

### Preparation of protein extracts of T-REx cells and their subcolonies

After washing with chilled HBS, cells on a 100-mm dish were lysed by adding 0.5 ml of lysis buffer (20 mM Hepes-NaOH, pH 7.5, 150 mM NaCl, designated L-buffer) supplemented with both inhibitors of proteinases and phosphatases, then cells were collected with a cell scraper (17-mm width). The suspension was transferred into a 1.5-ml tube, sonicated by a Bioruptor (BM Equipment) in an ice-water bath (15 × 10-s pulses at 10-s intervals), and centrifuged (25000 × ***g***, 30 min, 4°C, Kubota 3780, rotor AF-2536A). The supernatants were aliquoted, snap-frozen in liquid nitrogen, and stored at −80°C until use. The obtained pellets were dissolved in 50 μl of lysis buffer containing SDS (1% SDS, 1 mM EDTA in 10-mM Hepes-NaOH, pH 7.5, designated LS-buffer), sonicated, and centrifuged (10000 × ***g***, 1 min, 4°C. After measurement of the protein concentration, the sample was boiled in SDS/PAGE sample buffer.

### Isolation of Flag-mBcnt using anti-Flag antibody-conjugated agarose beads

The frozen supernatant of the subcolony #1 cells was thawed, and Nonidet P-40 (NP-40) was added to a final concentration of 0.05%. After sonication for 30 s in an ice-water bath followed by centrifuging at 10000 × ***g*** for 1 min at 4°C, the supernatant (1.2 mg in 1 ml) was mixed with anti-Flag-conjugated agarose beads (20 μl settled volume) in a 1.5-ml siliconized tube and incubated in a rotary shaker for 2 h, at 4°C. The mixture was centrifuged for 30 s, and the supernatant was saved as the unbound fraction. The pellet was resuspended in 100 μl of L-buffer containing 0.05% NP-40 plus inhibitors and transferred to a spin column using a 200-μl wide-bore tip. The tube was once more washed with L-buffer plus NP-40, and the suspension was recovered to the spin column. The flow-through fraction obtained by centrifugation was saved as the first wash fraction. The protein-bound agarose was washed twice with the same buffer, followed by washing once with HBS, and then the bound proteins were eluted by incubating with 50 μl of Flag peptide solution (150 μg in HBS) at 4°C for 30 min (Eluate #1) and another 5 min (Eluate #2), sequentially. The agarose was further treated with 50 μl of glycine-HCl (50 mM, pH 2.5), and its eluate and the agarose in the column were immediately neutralized with 2 M Tris. Finally, 50 μl of 1 × SDS/PAGE sample buffer was added to the column, vortexed, and boiled for 5 min. All of the fractionated samples except the fraction eluted with SDS/PAGE sample buffer, were boiled in 1 × SDS/PAGE sample buffer after being adjusted with 4 × SDS/PAGE buffer. Each sample was separated on 12.5 % SDS/PAGE and followed by western blot analysis using anti-Flag Ab. For LC-MS/MS analysis, Eluate #1 and Eluate #2 described above (each ∼40 μl) were concentrated with acetone according to a protocol (http://tools.thermofisher.com/content/sfs/brochures/TR0049-Acetone-precipitation.pdf). The dry pellet was once dissolved in 6 μl of LS-buffer, and 2 μl of 4 × SDS/PAGE sample buffer was added, followed by boiling for 3 min. After separating the sample on 12.5% gel SDS/PAGE 10 min longer than usual to ensure the separation of the upper and lower bands, the gel was fixed with 50% MeOH-10% acetic acid solution, stained with 0.25% CBB, and then de-stained in 10% MeOH-7% acetic acid solution.

### Preparation of protein extracts from mouse and rat brain

The whole brain and cerebellum were dissected from C57BL/6J mice (P0, male) and Wistar rats (P0-P56, male), respectively, after euthanasia by diethyl ether anesthesia. These samples were snap-frozen in liquid nitrogen and stored at −80°C until use. Frozen samples were crushed with a hammer on dry ice and immediately transferred to a glass-Teflon homogenizer containing LS-buffer (1 ml per 100 mg of samples). Then, tissues were homogenized at 600 rpm using a digital homogenizer and boiled for 5 min. The homogenates were sonicated (12 × 10-s pulses at 20-s interval) and centrifuged (28000 × ***g***, 30 min, 20°C). In a case of a mouse brain of the embryonic day around 17, a frozen piece (∼40 mg) was wrapped with aluminum foil, crushed with a plier cooled with liquid nitrogen, transferred to a 1.5-ml Bio Masher, and soaked in 200 μl of chilled LS-buffer. Then, the tissues were homogenized cooling in ice, boiled for 5 min, sonicated, and centrifuged (15000 × ***g***, 10 min). The supernatants were used for Western blot analysis. The protein concentrations of the supernatants were estimated using a Bicinchoninic Acid protein assay kit (BCA kit) with bovine serum albumin as a standard.

### Enrichment of bovine Bcnt/Cfdp1 using Phos-tag agarose

Bovine placenta (Holstein on 116th day of the pregnancy) as small frozen pieces, which were a gift from Dr Kazuyuki Hashizume (Iwate University, Morioka), were stored in liquid nitrogen until use. Tiny frozen tissue was obtained by cutting the pieces with a knife (∼30 mg); then, they were further minced with a razor blade, and proteins were extracted in the following two ways. The minced pieces were placed in a 1.5-ml BioMasher, soaked in 200 μl of cold L-buffer plus inhibitors, homogenized, and then mixed with 10 μl of 20% SDS, followed by boiling for 5 min. The extract was sonicated for 2.5 min, centrifuged (15000 × ***g***, 10 min, 22°C), and the supernatant was used as a whole extract. For enrichment of Bcnt/Cfdp1 content, the minced tissue pieces in the 1.5-ml BioMasher were homogenized in 100 μl of chilled RIPA buffer (20 mM Tris-HCl, pH 7.5, 150 mM NaCl, 0.5% sodium deoxycholate, 1% NP-40, 1 mM EDTA) plus both inhibitors of proteinases/phosphatases. After washing with another 100 μl of RIPA buffer, the suspension was centrifuged (15000 × ***g***, 10 min, 4°C) and the supernatant was aliquoted, snap-frozen in liquid nitogen and stored in −80°C until use. After dilution of the supernatant with RIPA buffer to 200 μg/100 μl, the suspension was mixed with Phos-tag agarose (100 μl settled volume) in a spin column, and the protein-bound beads were washed three times with each 200 μl of washing buffer and eluted three times with each 100 μl of elution buffer per tube according to the manufacturer’s protocol. Each eluate was precipitated with TCA and washed twice with acetone according to a protocol. After heating at 95°C, 50 μl of 1 × SDS/PAGE sample buffer was added and solubilized by a mixer (Tomy MT-360) for the Western blot analysis. Bcnt/Cfdp1 content in the above pellets (PPT) was estimated by adding 6.7 μl of 4 × SDS/PAGE sample buffer to PPT (∼20 μl), boiling for 5 min, and sonicating for 2.5 min. For larger scale preparation, the above 700 μg of the extract was used.

### Immunoblotting

Procedures of SDS/PAGE using 12.5% or 15% gel and blotting onto membranes were virtually the same as previously described [16,18]. Abs against Bcnt/Cfdp1 are listed in Table 1. All immunoreactivity was visualized by a chemiluminescence method using horseradish peroxidase (HRP)-conjugated secondary Abs as described previously [18], except for using HRP-conjugated goat anti-mouse IgG light chain instead of anti-mouse IgG (H+L).

### Mass spectroscopy analysis

The upper and lower bands of a doublet of F-mBcnt, which were detected by CBB staining, were separately cut out from the gel and digested with API, AspN, and chymotrypsin. Each digest was analyzed by nano-LC-MS/MS using a Q Exactive mass spectrometer, virtually the same as described previously [16]. Peptides derived from the upper and lower bands were quantified by a label-free quantification method using Proteome Discoverer Ver 2.2.0.388 (Thermo Fisher Scientific). The mass spectrometry proteomics data have been deposited to the ProteomeXchange Consortium via the PRIDE [19] partner repository with the dataset identifier PXD019016 and 10.6019/PXD019016.

### Isolation of total RNA

Frozen ES cells were homogenized with TRI reagent, and total RNAs were purified using PureLink RNA Mini kit according to the manufacturer’s protocol. Purified total RNAs were treated with TURBO DNase to eliminate contaminating genomic DNA, extracted with phenol/chloroform/isoamyl alcohol (pH 5.2), and re-purified using RNA Clean & Concentrator -25 kit. The concentration of the total RNA was determined by the absorbance at 260 and 280 nm using NanoDrop One (Thermo Fisher Scientific), and the quality was estimated using a 2100 Bioanalyzer (Agilent). RNA sequencing was outsourced using each total RNA from Cfdp1-K1 and vdR2-4, respectively (A269/280: 2.07 and 2.03; RIN: 9.9 and 9.6∼9.9, respectively) (Macrogen Japan Corp., Kyoto).

### Reverse transcription-PCR, plasmid construction, and PCR product analysis

cDNAs were synthesized from purified total RNAs of Cfdp1-K1, vdR2-4 cells, and whole mouse brain of a P56 C57BL/6J male by Superscript III First-Strand Synthesis SuperMix according to the manufacturer’s protocol using oligo-(dT) 20. The full-length ORF of m*Bcnt* or the fragment of m*Bcnt* exons 1-5 fused with *Hph* (hygromycin phosphotransferase) was amplified from each cDNA (1 ng of total RNA) by PCR using KAPA HiFi HotStart DNA polymerase, and PCR products were analyzed as described previously [18]. The primer sequences for detection of the gene trapped m*Bcnt* are 5′-gagctcggatccgccgccatggaggaattcgactccgaag-3′ (*Sac*I-*Bam*HI-m*Bcnt*, forward) and 5′-gagctcctcgagccgatgcaaagtgccgataaac-3′ (*hph-Xho*I-*Sac*I, reverse), respectively.

### Transcriptome analysis

The following is a summary of the report from Macrogen Corp Japan (Supplementary Appendix S1). The two cDNA libraries from the RNA of Cfdp1-K1 or dvR2-4 cells were prepared, and their sequences were obtained. The trimmed paired-end reads (read length 101) of 45,262,214 from Cfdp1-K1 or 52,245,252 from vdR2-4 were mapped to a mouse reference genome (UCSC GRCm38.p4/mm10, annotation RefSeq_2017_06_12).

## Results

### Detection of Flag-tagged mBcnt as a doublet band with differential phosphorylation

To characterize mammalian endogenous Bcnt/Cfdp1, we previously expressed exogenous His-hBCNT/CFDP1 as a reference. However, proteins that crossed the anti-His tag Ab provided a relatively high background, making it difficult to assign the signals of the endogenous target in the Western blot [16]. This time, as another reference, we expressed F-mBcnt in T-REx cells.

mBcnt/Cfdp1 is composed of 295 amino acids (aa), which is 4 aa less than the human counterpart (Figure 1, top panel, [1]). While 82 aa sequences of both BCNT-C domains are identical except for two aa residues, the N-terminal region has low homology between mouse and human (75%) and can be used as species-specific immunogens. T-REx cell colonies constitutively expressing F-mBcnt were isolated using G418 selection. Both the number and size of the antibiotic-resistant colonies expressing F-mBcnt were significantly lower and smaller than those expressing F-MCS as a control. The extracts from several colonies of each transfectant were prepared and evaluated with either anti-Flag Ab or anti-BCNT-C Ab (Table 1) using Western blot analysis. The signal with anti-Flag Ab showed no or two bands, whereas anti-BCNT-C Ab detected single or three bands (Figure 1A; Supplementary Figure S1A and B).

When comparing the doublet pattern between transiently expressing cells and constitutively expressing cells, the upper band of the transient expression was significantly stronger than that of the constitutive expression (Figure 1A). These features are similar to those of His-hBCNT/CFDP1, as previously reported [16].

To reveal the molecular difference between the upper and lower bands, they both were isolated from lysates of T-REx-derived colony #1, which constitutively express F-mBcnt, using anti-Flag Ab-conjugated agarose beads (Supplementary Figure S2 and Figure 1B). Each band excised from the gel was digested with three different proteases, and each digest was subjected to LC-MS/MS analysis (PXD019016 and 10.6019/PXD019016). The ratios of the upper to lower bands for each set of fragments and their phosphorylation sites are systematically presented in Supplementary Table S1 and Figure S3. The upper band is much more phosphorylated than the lower band. In particular, serine 246th mBcnt/Cfdp1, which corresponds to S250 of hBCNT/CFDP1 [16], is a preferential site of phosphorylation.

### Evaluation of anti-BCNT-C antibody

**Figure 2:**
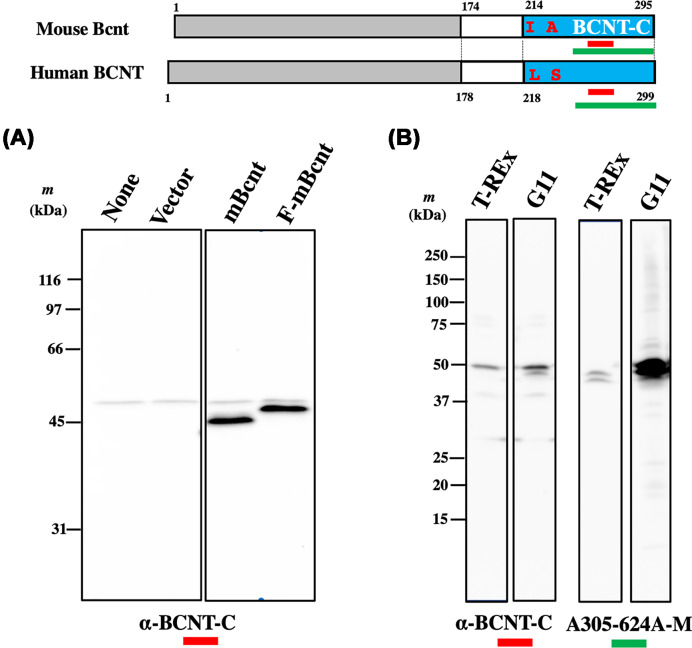
Comparative assessment of Western blot signals between two antibodies: anti-BCNT-C and A305-624A-M **Top panel**: Structures of human BCNT/CFDP1 (Human BCNT) and mouse Bcnt/Cfdp1 (Mouse Bcnt) are shown as in the top panel of Figure 1, and the location of each immunogen of anti-BCNT-C antibody (Ab) (red bar) or A305-624A-M (green bar) is presented. (**A**) Comparison of mobilities between Flag-tagged (F-mBcnt) and tag-free mouse Bcnt/Cfdp1 (mBcnt) on SDS/PAGE. HEK239T cells were transfected with Flag-MCS-pcDNA3.1 plasmid carrying m*Bcnt* or m*Bcnt* in BsrGI-MCS-pcDNA3.1 (Accession No. LC311017), and their extracts were subjected to Western blot analysis with anti-BCNT-C Ab. Left panel: None and Vector indicate untreated and a blank vector control, respectively. The results published in [18] were rearranged and shown. (**B**) The supernatants were prepared from each cell lysate of T-REx or G11 cells (His-hBCNT/CFDP1 expressing clone) by centrifugation. Equal amounts of protein (20 μg) were subjected to Western blot analysis with either anti-BCNT-C Ab (left two lanes) or A305-624A-M (right two lanes).

Based on the results of the Western blot analyses with anti-BCNT-C Ab [16], we previously presumed that the ∼50-kDa signal corresponded to endogenous hBCNT/CFDP1 in both T-REx cells and its G11 clone, the latter of which constitutively expressed exogenous His-hBCNT/CFDP1. F-mBcnt is 306 aa in length, which is 7 aa longer than hBCNT/CFDP1 and is similarly phosphorylated (Supplementary Table S1 and Figure S3). Furthermore, the effect of acid Flag tag (DYKDDDDK) on mobility in SDS/PAGE is more significant than that of the 11 aa diference (1.2 kDa) between F-mBcnt and mBcnt (Figure 2A and [18]). Thus, it should be larger than His-hBCNT/CFDP1. However, F-mBcnt appeared below the ∼50-kDa band (Figure 1). From these results, the ∼50-kDa is a band that is not likely derived from endogenous hBCNT/CFDP1.

Thus, we reexamined the previous Western blot data of His-hBCNT/CFDP1 [16] by introducing two other commercially available anti-Bcnt/Cfdp1 Abs: 26636-1-AP and A305-624A-M (Table 1). We chose these two Abs from the following criteria. The main antigen regions are from BCNT-C domain, and the candidate signal (s) is reported in a region that is significantly smaller than 50 kDa from the Western blot analysis. However, as we detected an extra ∼50 kDa signal in 26636-1-AP (shown below), we compared the western signal patterns of cell lysates between anti-BCNT-C Ab and A305-624A-M. Both Abs recognized exogenously expressed His-hBCNT/CFDP1 in the G11 extract but showed distinctly different patterns in the parental T-REx extract (Figure 2B). It is noteworthy that anti-BCNT-C Ab reacted to a band above the doublet detected with A305-624A-M. The difference between the two patterns was confirmed by reprobing with each of the replaced Abs (Supplementary Figure S4). Whereas anti-BCNT-C Ab revealed a few other bands, A305-624A-M detected mainly the doublet band; however, so far, no direct evidence suggests that it corresponds to the doublet of F-mBcnt shown in Figure 1. Since we confirmed the ability of A305-624A-M to immunoprecipitate His-hBCNT/CFDP1 using G11 cell extract and anti-His Ab, we inferred that the distinct ∼50-kDa band detected with the anti-BCNT-C Ab shown in Figure 1 and Supplementary Figure S1 is an off-target signal.

### Assignment of the candidate signal of mBcnt/Cfdp1 using ES mutant cells

**Figure 3:**
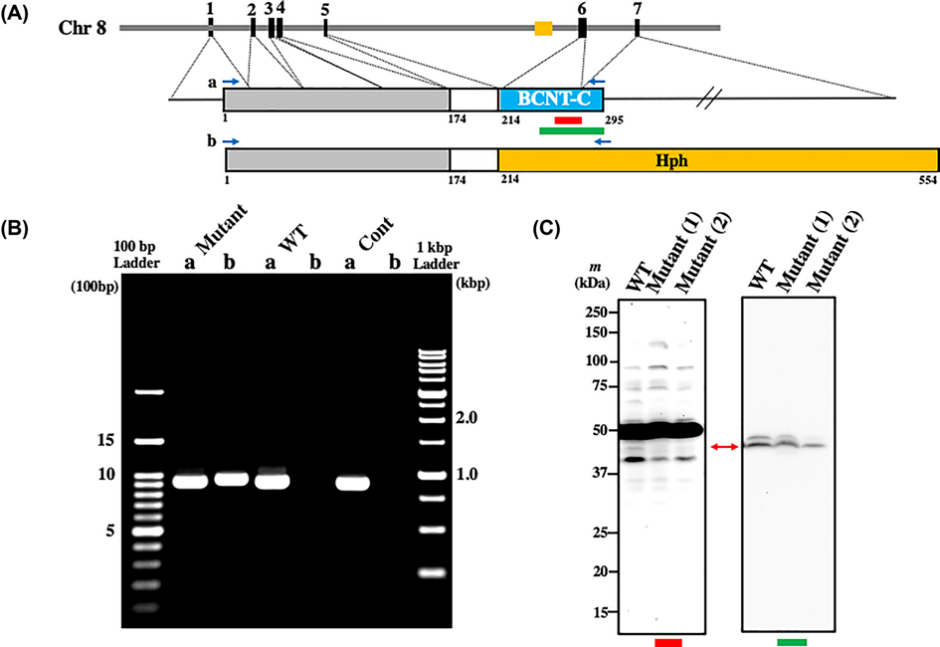
Assignment of the endogenous Bcnt/Cfdp1 signal using mutant ES cells by Western blot analysis (**A**) Location of the inserted gene trap vector in mouse *Bcnt/Cfdp1* and the expected fusion protein. The mouse *Bcnt/Cfdp1* gene is located in the chromosome 8 (Chr 8) and consists of 7 exons. Dashed lines indicate corresponding regions of each exon to Bcnt/Cfdp1*.* A brown box on Chr 8 indicates the gene trap vector. Red and green bars under Bcnt/Cfdp1 indicate each location of immunogens to raise anti-BCNT-C antibody (Ab) and A305-624A-M, respectively. At the top, the structure of expected fusion protein is shown, which is encoded by *Bcnt/Cfdp1* exons 1-5 and *Hph* (hygromycin phosphotransferase) derived from the gene trap vector. Four blue arrows at the top of each structure of the fusion protein and Bcnt/Cfdp1 indicate the location of two sets of PCR primers for RT-PCR analysis. (**B**) RT-PCR analysis of *Bcnt/Cfdp1* mRNA. Using each cDNA from Cfdp1-K1 (Mutant), its parental cells vdR2-4 (wild-type, WT), or mouse brain (Cont), RT-PCR was carried out. Lanes a and b indicate RT-PCR products corresponding to the full-length ORF of m*Bcnt/Cfdp1* (928 bp) (a) and the fused gene of m*Bcnt* exons 1-5 and a part of *Hph* in the gene trap vector (948 bp) (b), respectively. The PCR products and DNA size ladder markers were separated in agarose gel, followed by staining with ethidium bromide. (**C**) Assessment of Western blot signal of endogenous Bcnt/Cfdp1 using extracts of Cfdp1-K1 and its parental cells. Cell extracts from an equal number (∼2 × 10^5^ cells) of vdR2-4 (WT) and Cfdp1-K1 (Mutant) cells, latter which had been serially passaged in the presence [Mutant (2)] or absence [Mutant (1)] of G418/puromycin were subjected to Western blot analysis with either anti-BCNT-C Ab (left filter) or 305-624A-M (right filter). A red two-headed arrow indicates a candidate signal of the endogenous mouse Bcnt/Cfdp1.

To assign the endogenous Bcnt/Cfdp1 signal using Western blot analysis, we utilized a mouse embryonic stem (ES) cell line (Cfdp1-K1) listed as homozygous m*Bcnt/Cfdp1* mutant cells (i.e. double-knockout cell line) [20]. The gene trap vector is reported to be inserted in intron 5 of *mBcnt/Cfdp1* (Figure 3A) (GenBank: accession number AG999723.1). As BCNT-C is encoded by exons 6 and 7 (Figure 3A), the Cfdp1-K1 cell lysate can be used as a potential negative control (knockout cell extracts) to evaluate the Abs generated against antigens derived from BCNT-C if the cells are homozygous m*Bcnt/Cfdp1* mutants.

First, we examined by reverse transcription-polymerase chain reaction (RT-PCR) whether *Bcnt/Cfdp1* is knocked out in Cfdp1-K1. After subculturing in the presence or absence of G418 and puromycin, both of which can delete the feeder layer cells, we prepared cDNAs from Cfdp1-K1 (mutant) and its parental cells, vdR2-4 (wild-type). We then examined the expression of m*Bcnt/Cfdp1* mRNA by RT-PCR and DNA sequencing of the products. In the cDNA from Cfdp1-K1, we detected PCR products corresponding to the full-length *mBcnt/Cfdp1* ORF as well as the fusion gene coding *Bcnt/Cfdp1* exons 1-5 and *Hph* derived from the gene trap vector (Figure 3B). This result indicates that *Bcnt/Cfdp1* was not double knockout in Cfdp1-K1 cells, even though the gene trap vector was inserted adequately into intron 5. Further comparative transcriptome RNA sequencing between Cfdp1-K1 and vdR2-4 revealed that 694 genes were expressed with more than a 2-fold difference, while 188 genes were up-regulated, and 506 genes were down-regulated (Supplementary Appendix S1 and Table S2). Among them, *Bcnt/Cfdp1* mRNA in the Cfdp1-K1 cells was reduced to 74.4% to that of the parent cells (101.75 vs. 136.79 FPKM, the top row of Supplementary Table S3). Besides, the mRNA of the flanking genes *Bcar1/Cas* and *Tmem170* (https://www.ncbi.nlm.nih.gov/gene/23837) and several housekeeping genes frequently used as internal controls were also significantly altered (Supplementary Table S3).

Next, we examined whether anti-BCNT-C Ab and A305-624A-M can detect the differential expression of Bcnt/Cfdp1 between mutant and parental cells using western blot analysis. Of the several bands detected by anti-BCNT-C Ab, one band of ∼45-kDa (indicated by a red arrow in Figure 3C) was distinctively reduced in the Cfdp1-K1 cells compared with vdR2-4 cells, which was also observed with A305-624A-M. These results suggest that the ∼45-kDa band is a candidate for the endogenous mBcnt/Cfdp1 in mouse ES cells.

### Evaluation of the anti-mBcnt-N antibody and assignment of the mBcnt/Cfdp1 signal

**Figure 4:**
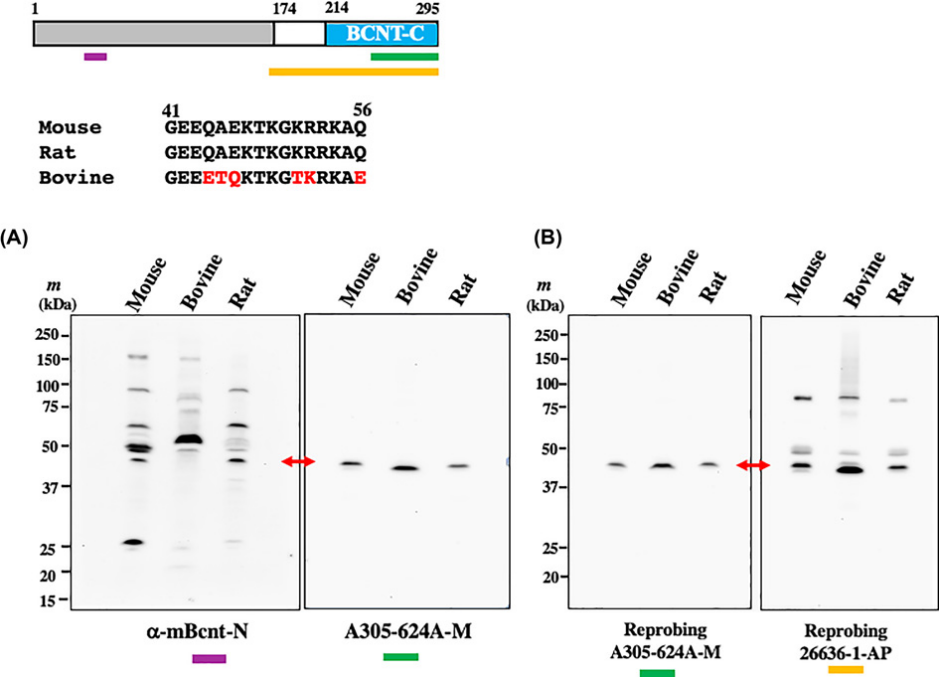
Validation of the anti-mBcnt-N antibody Top panel: Structure of mouse Bcnt/Cfdp1 and amino acid sequence alignment of the immunogen to raise the anti-mBcnt-N antibody. The structure of mBcnt/Cfdp1 is schematically shown. Three colored bars present the locations of each immunogens to generate the anti-mBcnt-N antibody (Ab) (purple), A305-624A-M (green), and 26636-1-AP (ocher), respectively. Middle panel: Alignment of the immunogen for the anti-mBcnt-N Ab generation and its counterparts of rat and bovine. Red letters indicate different amino acids from the immunogen peptide. (**A** and **B**) The specificity of anti-mBcnt-N Ab concerning a ∼45 kDa band. (A) Bovine placenta extract enriched for Bcnt/Cfdp1 content (20 or 30 μg), and brain extracts of mouse and rat (15 or 20 μg) were separated on SDS/PAGE, followed by Western blot analyses with either anti-mBcnt-N Ab (left filter, the larger amounts of protein, i.e. 20 or 30 μg) or A305-624A-M (right filter, the smaller amounts of protein, i.e. 15 or 20 μg). (B) Each filter was reprobed with A305-624A-M (left filter) and 26636-1-AP (right filter), respectively. Two red two-headed arrows indicate the candidate signals of the endogenous Bcnt/Cfdp1.

As mentioned above, the C-terminal region of BCNT members is highly conserved, but the N-terminal region is variable among species. To further investigate whether the ∼45-kDa band is a valid signal, an anti-mBcnt/Cfdp1 Ab was generated using a mouse N-terminal peptide as an immunogen (anti-mBcnt-N Ab, Table 1 and top panel of Figure 4). Rat Bcnt/Cfdp1 has the same sequence as the immunogenic peptide derived from mBcnt/Cfdp1, but the bovine counterpart has a distinctively different amino acid sequence (Figure 4, middle panel), suggesting that the cross-reactivity with the anti-mBcnt-N Ab was expected to be low. Thus, we can use tissue extracts of bovine and rat as a potential negative control and positive control, respectively, to evaluate the specificity of anti-mBcnt-N Ab against the endogenous mBcnt/Cfdp1 in the Western blot analysis. After concentrating the Bcnt/Cfdp1 content in the bovine extract with Phos-tag agarose [21] to enrich the phosphorylated proteins (Supplementary Figure S5), we showed that anti-mBcnt-N Ab detected the ∼45-kDa signal in mice and rats but not in cattle (Figure 4A, left filter), while A305-624A-M detected the ∼45-kDa signal in all tissue extracts (Figure 4A, right filter). This result strongly suggests that anti-mBcnt-N Ab specifically recognizes endogenous mBcnt/Cfdp1 despite nonspecific cross-reactions with multiple unknown proteins. We further confirmed that the ∼45-kDa signal was a valid target signal using another anti-hBCNT/CFDP1 Ab, 26636-1-AP (Figure 4B, right filter).

**Figure 5:**
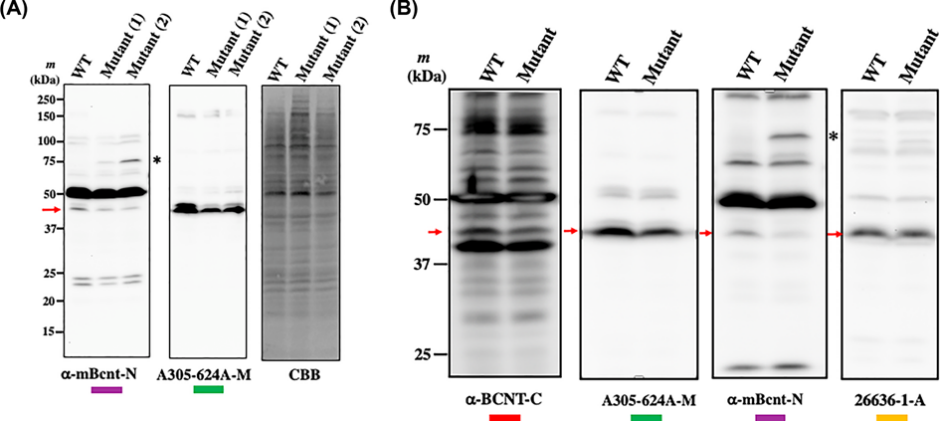
Assignment of the mouse Bcnt/Cfdp1 signals by Western blot analysis (**A**) Equal amounts of cell extract (20 μg protein) of vdR2-4 (WT) and Cfdp1-K1 (Mutant) cells, latter which were serially passaged in the presence [Mutant (2)] or absence [Mutant (1)] of G418/puromycin, were subjected to a Western blot analysis with anti-mBcnt-N antibody (Ab) (left filter) or A305-624A-M (middle filter). A red arrow indicates a signal of mouse Bcnt/Cfdp1. A filter was stained with Coomassie Brilliant Blue (CBB) to check the amounts of loading proteins (right filter). (**B**) Four filters were prepared as the same as (**A**) but without [Mutant (1)], and Western blotting was performed with four anti-Bcnt/Cfdp1 Abs used in the present study shown below each filter. Red arrows indicate signals of mouse Bcnt/Cfdp1. A band detected with the anti-mBcnt-N Ab at ∼75 kDa (shown as *) is probably the fusion protein of a part of mBcnt/Cfdp1 (exon 1-5) and hygromycin phosphotransferase, as schematically shown at the top of Figure 3A, which has a calculated molecular mass of 63.9 kDa, but may run slowly on SDS/PAGE due to the acid stretch located in the N-terminal region of the mouse Bcnt/Cfdp1 [16].

Using anti-mBcnt-N Ab or A305-624A-M, we performed Western blot analysis with two kinds of Cfdp1-K1 cell lysates prepared after passages in the presence or absence of G418 / puromycin (Figure 5A). The results showed that both Abs exhibited a ∼45-kDa band with an intensity that is distinctively reduced in both Cfdp1-K1 lysates as compared with that in the parental cell lysate. Finally, we confirmed that all four anti-Bcnt/Cfdp1 Abs used in the present study exhibited reasonable signals of ∼45-kDa (Figure 5B). From these results, we conclude that the ∼45-kDa signal corresponds to the endogenous mBcnt/Cfdp1.

### Expression of mBcnt/Cfdp1 in the early stage of brain development

**Figure 6:**
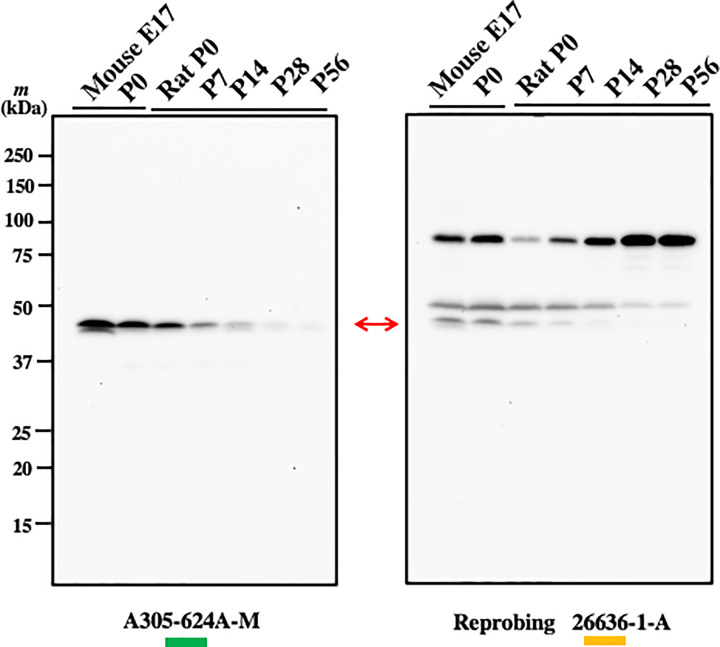
Preferential expression of Bcnt/Cfdp1 in the early developmental stages of mouse and rat brains Mouse brain extracts of approximately embryonic day17 (E17) and postnatal day 0 (P0), and of rat cerebral extracts from postnatal day samples (denoted by P# on the top of each lane) were loaded (each 20 μg amounts of protein) and subjected to a Western blotting analysis with A305-624A-M (left panel). The filter was stripped and reprobed with 26636-1-AP (right panel). A red two-headed arrow indicates the signals of the endogenous Bcnt/Cfdp1.

RNA profiling data of mouse and rat (https://www.ebi.ac.uk/gxa/home) show that Bcnt/Cfdp1 mRNA expresses ubiquitously and preferentially in the early stage of development. However, recent studies have revealed pervasive discordance between mRNA levels and protein levels, especially in embryonic development [15]. Therefore, we examined Bcnt/Cfdp1 expression during the developmental stages of mouse brain and rat cerebrum using the evaluated anti-Bcnt/Cfdp1 Abs described above. The results showed that Bcnt/Cfdp1 preferentially expresses in the early stages and significantly decreased according to the postnatal stages in the rat cerebrum (Figure 6).

## Discussion

In the present study, we assigned the signal of the endogenous mBcnt/Cfdp1 as a ∼45-kDa protein using Western blot analysis by utilizing various target-related materials, and we showed that mouse and rat Bcnt/Cfdp1 are expressed preferentially at early stages of brain development. Moreover, based on the problems encountered during the present study, we discuss immune-cross reactions with off-target proteins.

We generated F-mBcnt-expressing cells instead of those of His-hBCNT/CFDP1 because the latter turned out to be unsuited to assign the endogenous hBCNT/CFDP1 band due to the difficulty to distinguish anti-BCNT-C Ab positive signals from the high background of the anti-His tag Ab positive proteins [16] such as Nono (p54rnb), which contains the HHQHHH sequence in the N-terminus region (NP_001138880) (Supplementary Figure S6). F-mBcnt appeared as a doublet using Western blot analysis, and the upper band was much more phosphorylated than the lower band. In particular, the phosphorylation of the Ser^246^ residue in the WESF motif of BCNT-C domain was characteristic. The doublet of mBcnt/Cfdp1 was probably caused by the presence or absence of Ser^246^ phosphorylation as shown in hBCNT/CFDP1 [16] because the characteristic phosphorylation is similar [16]. However, the ratio of the upper and lower bands was variable, and the reason for this is currently unknown.

We first evaluated a custom-made anti-BCNT-C Ab that detected an extra band in F-mBcnt-expressing cell lysates (Figure 1 and Supplementary Figure S1). We initially presumed that the band was hBCNT/CFDP1. We used A305-624A-M, which was confirmed for immunoprecipitation ability using the His-hBCNT/CFDP1-expressing G11 cell extract with anti-His tag Ab (data not shown). We compared the immune-positive signals detected with ant-BCNT-C Ab and A305-624-M and found that their patterns were distinctively different in the parental cell extract, whereas both Abs recognized the exogenous hBCNT/CFDP1 in the extract of the G11 clone (Figure 2B). Therefore, we utilized a Cfdp1-K1 cell line that was listed as a mouse *Bcnt/Cfdp1* homozygous mutant. The cell is a product of a library of random mutations introduced by gene trap vector insertion in Bloom-deficient ES cells and was selected for populations of homozygous mutant cells following mitotic recombination [20]. However, RT-PCR analysis of the cDNA from Cfdp1-K1 revealed the presence of mRNA corresponding to the full-length ORF of the m*Bcnt/Cfdp1*. Indeed, a comparative analysis of the transcriptome RNA sequencing results showed that *Bcnt/Cfdp1* mRNA levels of Cfdp1-K1 were 74.4% of its parental cells. Splicing may efficiently occur by skipping the acceptor site in the gene trap vector, which is located in the intron 5 that spans over 50 kb (Figure 3A). Although Cfdp1-K1 was not a double-knockout cell line of m*Bcnt/Cfdp1*, it was useful as a m*Bcnt/Cfdp1* knockdown cell to select a promising candidate signal of mBcnt/Cfdp1 at ∼45 kDa.

On the other hand, an attempt was made to generate *Bcnt/Cfdp1* knockout MDCK (Madin-Darby Canine Kidney) cells by targeting its exon 1 by CRISPR-Cas9 technology as in production of β- and γ-catenin double knockout cells [22]; the expected cells were not obtained so far (W. Kobayashi, personal communication). By using Cfdp1-K1 and its parental cells, we were finally able to assign the signal of endogenous mBcnt/Cfdp1 detected by two Abs raised against unrelated immunogens: a mouse N-terminal peptide and a peptide derived from BCNT-C domain, the latter of which is highly conserved in mammalian Bcnt/Cfdp1.

The following pieces of evidence show that the ∼45-kDa band is the signal of endogenous Bcnt/Cfdp1 using Western blot analysis. First, among several signals detected by anti-BCNT-C Ab, the ∼45-kDa signal was distinctively reduced in the Cfdp1-K1 cell extract compared with the signal of the parental cell extract (Figures 3C and 5). Second, the differential ∼45-kDa signal was also detected by three other antibodies for Bcnt/Cfdp1 (i.e. anti-mBcnt-N Ab, A305-624A-M, and 26636-1-AP), two of which were generated using mutually unrelated immunogens (Figures 4 and 5). We confirmed the immunoprecipitation ability of A305-624A-M among these Abs. More importantly, we validated the specificity of the anti-mBcnt-N Ab concerning the ∼45-kDa signal using a potential negative control by preparation of enriched Bcnt/Cfdp1 content in the bovine tissue with Phos-tag agarose (Figure 4 and Supplementary Figure S5).

The apparent molecular size of the target band (∼45-kDa) on SDS/PAGE appears significantly smaller than those of signals reported by many available anti-Bcnt/Cfdp1 Abs, including anti-BCNT-C Ab. The molecular behavior of the ∼50-kDa band(s) was significantly different from that of endogenous Bcnt/Cfdp1; therefore, the 50-kDa protein(s) is probably an off-target(s). Our initial conjectures based on the results using anti-BCNT-C Ab in our previous articles are all misdirected, including conjectures on the 50-kDa signal (refer to Abstract in [16]), the 43-kDa signal (glutamine synthetase, [18] refer to Figure 3C in [17] and Figure 1B in [23]), and the intracellular localization of Bcnt/Cfdp1 by immunostaining (refer to Figure 6B in [24]).

We recently introduced two-dimensional gel electrophoresis for the separation of Cfdp-K1 cell extracts and detected a broad smear and doublet bands around 45 kDa with an expected isoelectronic point using A305-624-M (Calculated IP value: 4. 80, Iwashita and Nakashima, unpublished data). On the other hand, we did not detect two bands at ∼37 and ∼19 kDa with A305-624A-M, which is shown in its catalog.

It was evident that anti-BCNT-C Ab detected a weak signal to endogenous Bcnt/Cfdp1 with many proteins that are distinct for the target (Figures 3C and 5). A similarly severe situation that Abs have no ability to detect the endogenous target protein due to many nonspecific bands have been reported (e.g. [25,26]). Concerning the troublesome evaluation of anti-Bcnt/Cfdp1 Abs, which has caused inappropriate assignments using Western blot analysis, several factors could be avoided, while others would be difficult to avoid. First, the expression information of Bcnt/Cfdp1 mRNA should be used more efficiently. It is highly expressed in mouse embryo brains, which is much more suitable than adult samples to identify the target and to evaluate anti-Bcnt/Cfdp1 Abs. Second, although other independent anti-Bcnt/Cfdp1 Abs were required and had been prepared using such as the N-terminal region as immunogens, no Abs was obtained, which satisfied the criteria of a single band at the appropriate molecular range. However, it is not necessary to follow single-band criteria, if we have appropriate information concerning the interest target(s) in the Western blotting analysis. In spite of many nonspecific proteins (Figure 4), we were able to show the specificity of anti-mBcnt-N Ab concerning the target ∼45 kDa band. Many similar cases such as anti-Rho Ab [27] have been reported. Third, although nonspecific proteins are generally various, which are recognized by multiple Abs raised against different immunogens of the same target, a relatively strong signal(s) near 50-kDa seemed to be commonly detected by many available anti-Bcnt/Cfdp1 Abs including anti-BCNT-C Ab and 26636-1-AP, resulting in an incorrect assignment. The second and third points may be related to the properties of Bcnt/Cfdp1, which mainly consists of structurally disordered regions. It is noteworthy that the epitopes from the antigen of disordered regions are smaller than those of the ordered regions and that the former epitopes interact more efficiently with their Abs [28]. Thus, the commonly available anti-Bcnt/Cfdp1 Abs that have been consequently generated against immunogens of its structurally disordered regions may cause cross-reactions with various proteins with high affinity, resulting in the poor quality of Ab. Fourth, complex migration of Bcnt/Cfdp1 on SDS/PAGE made it challenging to assign a valid signal. hBCNT/CFDP1 as well as mBcnt/Cfdp1 is expressed as a doublet band and migrates more slowly on SDS/PAGE than expected from the calculated molecular mass (33.6 kDa of hBCNT/CFDP1 and 32.7 kDa of mBcnt/Cfdp1). This feature may be mainly due to the acid stretch of the N-terminal region and the Ser phosphorylation of BCNT-C domain [16].

Off-target problems have been widely discussed in many Ab validation studies (e.g. [25,26]), including specific in-depth efforts for Ab evaluation (e.g. [29]). Moreover, a strategy for Ab validation has been proposed [30]. However, the focus of this discussion appears to be blurred, at least regarding Western blot analysis. Abs do not act under the confined all-or-nothing modes via specific reactions. It is noteworthy that Abs recognize target molecules based on their physicochemical environment, which is the chemical entity of the epitope and does not necessarily correspond to the linear amino acid sequence [18,31,32]. For example, the following two cases reflect topological or stereochemical similarity of spatially limited environments that determine the common epitopes between completely different proteins: Bcnt/Cfdp1 and glutamine synthetase [18], and phosducin and β-actin [33], respectively.

Although Western blot analysis is much more powerful to examine the target proteins of extracts than mass spectroscopy analysis, these tools are fundamentally different—that is, individual Ab is not a tool to identify a molecule but rather a tool for checking inconsistencies such as with differential tissue distribution of mRNA expression. The specificity of an Ab depends largely on the relative expression level of the target molecule. Therefore, it is critical to understand the efficacy and limitations of the Ab used in any experiments.

Recently, mass analysis tools have become remarkably advanced and widely available. Thus, it is now much easier to identify molecules that have been considered to be false targets due to nonspecific cross-reaction with Abs. These trials to identify the off-targets may provide byproducts of the excellent Abs, such as conformation-specific antibodies against the proteins.

As a more reliable anti-Bcnt/Cfdp1 Ab (e.g. A305-624A-M) becomes available, it is then possible to more accurately characterize endogenous Bcnt/Cfdp1, including subcellular localization and tissue distribution. On the other hand, the comparative transcriptome analysis of Cfdp1-K1 cells revealed that only a 25% decrease in Bcnt/Cfdp1 mRNA resulted in a marked up-regulation or down-regulation of many genes (Supplementary Tables S2 and S3). The finding is consistent with a report by Messina that suggests that extensive effects on vital cellular functions were caused by RNAi-depletion of *Bcnt/Cfdp1* in HeLa cells [34].

Recently, the budding yeast Swc5 has been clearly shown to preferentially bind to Histone H2A via the tandem DEF/Y motifs in the N-terminus [35], while an additional regulatory factor(s) might be involved in vertebrate Bcnt/Cfdp1. This result implicates that the broad effect of Bcnt/Cfdp1 deficit caused by RNAi-depletion [34] or shown as in genome-mediated knockdown of Cfdp1-K1 cells may be directly caused by impairing the histone exchange reaction of chromatin remodeling complex. However, the natural genetic variation also indicates that extensive abnormal expression of mRNA, antisense and noncoding RNAs may be caused by a mutation in the fission yeast *Swc5* that does not encode the DEF/Y motif in the N-terminus [14]. To further elucidate the functional role(s) of Bcnt/Cfdp1, comprehensive analyses are required not only—at the protein levels with more appropriate Abs but also at RNA and DNA levels—using different materials, such as knockdown and knockout cells or mice and further disease model animals.

## Supplementary Material

Supplementary Figures S1-S6

Supplementary Tables S1-S4

Supplementary Appendix S1

## Abbreviations

Ab: antibody
anti-BCNT-C Ab: Ab generated using a peptide of 18 amino acid residues located in BCNT-C
BCNT-C: The C-terminal region of Bcnt/Cfdp1
Bcnt/Cfdp1: Bucentaur/Craniofacial developmental protein 1
F-mBcnt: Flag-tagged mouse Bcnt/Cfdp1
hBCNT/CFDP1: human BCNT/CFDP1
His-hBCNT/CFDP1: His-tagged hBCNT/CFDP1
mBcnt/Cfdp1: mouse Bcnt/Cfdp1
MCS: multi cloning site in a vector
SDS/PAGE: SDS-polyacrylamide gel electrophoresis
T-REx cell: a HEK293 cell derivative
WT: wild-type

## References

[1] Messina G., Celauro E., Atterrato M.T., Giordano E., Iwashita S. and Dimitri P. (2015) The Bucentaur (BCNT) protein family: a long-neglected class of essential proteins required for chromatin/chromosome organization and function. Chromosoma 124, 153–162 10.1007/s00412-014-0503-8 25547403

[2] Havugimana P.C., Hart G.T., Nepusz T., Yang H., Turinsky A.L., Li Z. et al. (2012) A census of human soluble protein complexes. Cell 150, 1068–1081 10.1016/j.cell.2012.08.011 22939629 PMC3477804

[3] Krogan N.J., Keogh M.C., Datta N., Sawa C., Ryan O.W., Ding H. et al. (2003) A Snf2 family ATPase complex required for recruitment of the histone H2A variant Htz1. Mol. Cell 12, 1565–1576 10.1016/S1097-2765(03)00497-0 14690608

[4] Mizuguchi G., Shen X., Landry J., Wu W.H., Sen S. and Wu C. (2004) ATP-driven exchange of histone H2AZ variant catalyzed by SWR1 chromatin remodeling complex. Science 303, 343–348 10.1126/science.1090701 14645854

[5] Nguyen V.Q., Ranjan A., Stengel F., Wei D., Aebersold R., Wu C. et al. (2013) Molecular Architecture of the ATP-Dependent Chromatin-Remodeling Complex SWR1. Cell 154, 1220–1231 10.1016/j.cell.2013.08.018 24034246 PMC3776929

[6] Wu W.H., Alami S., Luk E., Wu C.H., Sen S., Mizuguchi G. et al. (2005) Swc2 is a widely conserved H2AZ-binding module essential for ATP-dependent histone exchange. Nat. Struct. Mol. Biol. 12, 1064–1071 10.1038/nsmb1023 16299513

[7] Tramantano M., Sun L., Au C., Labuz D., Liu Z., Chou M. et al. (2016) Constitutive turnover of histone H2A.Z at yeast promoters requires the preinitiation complex. Elife 5, pii: e14243 10.7554/eLife.14243 PMC4995100 27438412

[8] Lin C.L., Chaban Y., Rees D.M., McCormack E.A., Ocloo L. and Wigley D.B. (2017) Functional characterization and architecture of recombinant yeast SWR1 histone exchange complex. Nucleic Acids Res. 45, 7249–7260 10.1093/nar/gkx414 28499038 PMC5499540

[9] Morillo-Huesca M., Clemente-Ruiz M., Andújar E. and Prado F. (2010) The SWR1 histone replacement complex causes genetic instability and genome-wide transcription misregulation in the absence of H2A.Z. PLoS ONE. 5, e12143 10.1371/journal.pone.0012143 20711347 PMC2920830

[10] Sun L. and Luk E. (2017) Dual function of Swc5 in SWR remodeling ATPase activation and histone H2A eviction. Nucleic Acids Res. 45, 9931–9946 10.1093/nar/gkx589 28973436 PMC5622370

[11] Messina G., Damia E., Fanti L., Atterrato M.T., Celauro E., Mariotti F.R. et al. (2014) Yeti, an essential *Drosophila melanogaster* gene, encodes a protein required for chromatin organization. J. Cell Sci. 127, 2577–2588 10.1242/jcs.150243 24652835

[12] Ohta S., Bukowski-Wills J.C., Sanchez-Pulido L., Alves F.L., Wood L., Chen Z.A. et al. (2010) The protein composition of mitotic chromosomes determined using multiclassifier combinatorial proteomics. Cell 142, 810–821 10.1016/j.cell.2010.07.047 20813266 PMC2982257

[13] Morozov V.M., Giovinazzi S. and Ishov A.M. (2017) CENP-B protects centromere chromatin integrity by facilitating histone deposition via the H3.3-specifne chaperone Daxx. Epigenetics Chromatin 10, 63 10.1186/s13072-017-0164-y 29273057 PMC5741900

[14] Clément-Ziza M., Marsellach F.X., Codlin S., Papadakis M.A., Reinhardt S., Rodríguez-López M. et al. (2014) Natural genetic variation impacts expression levels of coding, non-coding, and antisense transcripts in fission yeast. Mol. Syst. Biol. 10, 764 10.15252/msb.20145123 25432776 PMC4299605

[15] Liu Y., Beyer A. and Aebersold R. (2016) On the Dependency of Cellular Protein Levels on mRNA Abundance. Cell 165, 535–550 10.1016/j.cell.2016.03.014 27104977

[16] Iwashita S., Suzuki T., Yasuda T., Nakashima K., Sakamoto T., Kohno T. et al. (2015) Mammalian Bcnt/Cfdp1, a potential epigenetic factor characterized by an acidic stretch in the disordered N-terminal and Ser250 phosphorylation in the conserved C-terminal regions. Biosci. Rep. 35, pii: e00228 10.1042/BSR20150111 PMC4613681 26182435

[17] Iwashita S., Osada N., Itoh T., Sezaki M., Oshima K., Hashimoto E. et al. (2003) A transposable element-mediated gene divergence that directly produces a novel type bovine Bcnt protein including the endonuclease domain of RTE-1. Mol. Biol. Evol. 20, 1556–1563 10.1093/molbev/msg168 12832649

[18] Nakashima K., Iwashita S., Suzuki T., Kato C., Kohno T., Kamei Y. et al. (2019) A spatial similarity of stereochemical environments formed by amino acid residues defines a common epitope of two non-homologous proteins. Sci. Rep. 9, 14818 10.1038/s41598-019-51350-2 31616018 PMC6794283

[19] Perez-Riverol Y., Csordas A., Bai J., Bernal-Llinares M., Hewapathirana S., Kundu D.J. et al. (2019) The PRIDE database and related tools and resources in 2019: improving support for quantification data. Nucleic Acids Res. 47, D442–D450 10.1093/nar/gky1106 30395289 PMC6323896

[20] Horie K., Kokubu C., Yoshida J., Akagi K., Isotani A., Oshitani A. et al. (2011) A homozygous mutant embryonic stem cell bank applicable for phenotype-driven genetic screening. Nat. Methods 8, 1071–1077 10.1038/nmeth.1739 22020066

[21] Kinoshita-Kikuta E., Yamada A., Inoue C., Kinoshita E. and Koike E. (2011) A novel phosphate-affinity bead with immobilized Phos-tag for separation and enrichment of phosphopeptides and phosphoproteins. JIOMICS 1, 157–169

[22] Kobayashi W. and Ozawa M. (2018) The epithelial-mesenchymal transition induced by transcription factor LEF-1 is independent of β-catenin. Biochem. Biophys. Rep. 15, 13–18 29998192 10.1016/j.bbrep.2018.06.003 PMC6038150

[23] Iwashita S., Ueno S., Nakashima K., Song S.Y., Ohshima K., Tanaka K. et al. (2006) A tandem gene duplication followed by recruitment of a retrotransposon created the paralogous bucentaur gene (bcntp97) in the ancestral ruminant. Mol. Biol. Evol. 23, 798–806 10.1093/molbev/msj088 16384818

[24] Iwashita S., Nakashima K., Sasaki M., Osada N. and Song S.Y. (2009) Multiple duplication of the bucentaur gene family, which recruits the APE-like domain of retrotransposon: Identification of a novel homolog and distinct cellular expression. Gene 435, 88–95 10.1016/j.gene.2009.01.012 19393175

[25] Vanli G., Cuesta-Marban A. and Widmann C. (2017) Evaluation and validation of commercial antibodies for the detection of Shb. PLoS ONE 12, e0188311 10.1371/journal.pone.0188311 29194461 PMC5711028

[26] Qi W., Davidson B.A., Nguyen M., Lindstrom T., Grey R.J., Burnett R. et al. (2019) Validation of anti-glucocerebrosidase antibodies for western blot analysis on protein lysates of murine and human cells. Biochem. J. 476, 261–274 10.1042/BCJ20180708 30578288

[27] Jank T., Bogdanović X., Wirth C., Haaf E., Spoerner M., Böhmer K.E. et al. (2013) A bacterial toxin catalyzing tyrosine glycosylation of Rho and deamidation of Gq and Gi proteins. Nat. Struct. Mol. Biol. 20, 1273–1280 10.1038/nsmb.2688 24141704

[28] MacRaild C.A., Richards J.S., Anders R.F. and Norton R.S. (2016) Antibody Recognition of Disordered Antigens. Structure 24, 148–157 10.1016/j.str.2015.10.028 26712277

[29] McIntush E.W. (2013) Response: ’Antibody crossreactivity between the tumour suppressor PHLPP1 and the proto-oncogene β-catenin’. EMBO Rep. 14, 494–496 10.1038/embor.2013.68 23681440 PMC3674452

[30] Uhlen M., Bandrowski A., Carr S., Edwards A., Ellenberg J., Lundberg E. et al. (2016) A proposal for validation of antibodies. Nat. Methods 13, 823–827 10.1038/nmeth.3995 27595404 PMC10335836

[31] Caoili S.E. (2016) Expressing Redundancy among Linear-Epitope Sequence Data Based on Residue-Level Physicochemical Similarity in the Context of Antigenic Cross-Reaction. Adv. Bioinformatics 1276594 27274725 10.1155/2016/1276594 PMC4870339

[32] Dalkas G.A. and Rooman M. (2017) SEPIa, a knowledge-driven algorithm for predicting conformational B-cell epitopes from the amino acid sequence. BMC Bioinformatics 18, 95 10.1186/s12859-017-1528-9 28183272 PMC5301386

[33] Piotrowska U. and Adler G. (2010) Phosducin and monomeric β-actin have common epitope recognized by anti-phosducin antibodies. Immunol. Lett. 134, 62–68 10.1016/j.imlet.2010.08.010 20804785

[34] Messina G., Atterrato M.T., Prozzillo Y., Piacentini L., Losada A. and Dimitri P. (2017) The human Cranio Facial Development Protein 1 (Cfdp1) gene encodes a protein required for the maintenance of higher-order chromatin organization. Sci. Rep. 7, 45022 10.1038/srep45022 28367969 PMC5377257

[35] Huang Y., Sun L., Pierrakeas L., Dai L., Pan L., Luk E. et al. (2020) Role of a DEF/Y motif in histone H2A-H2B recognition and nucleosome editing. Proc. Natl. Acad. Sci. U.S.A. 117, 3543–3550 10.1073/pnas.1914313117 32001508 PMC7035559

