# Supplementary material for: Overcoming off-targets: assessing Western blot signals for Bcnt/Cfdp1, a tentative component of the chromatin remodeling complex
Source: Biosci Rep. 2020 Jun 9;40(6):BSR20194012. doi: 10.1042/BSR20194012 (PMC7284322; doi:10.1042/BSR20194012)
Supplement: Supplementary Figures S1-S6 [file BSR-2019-4012_supp.pdf]

Supplementary Figure S1

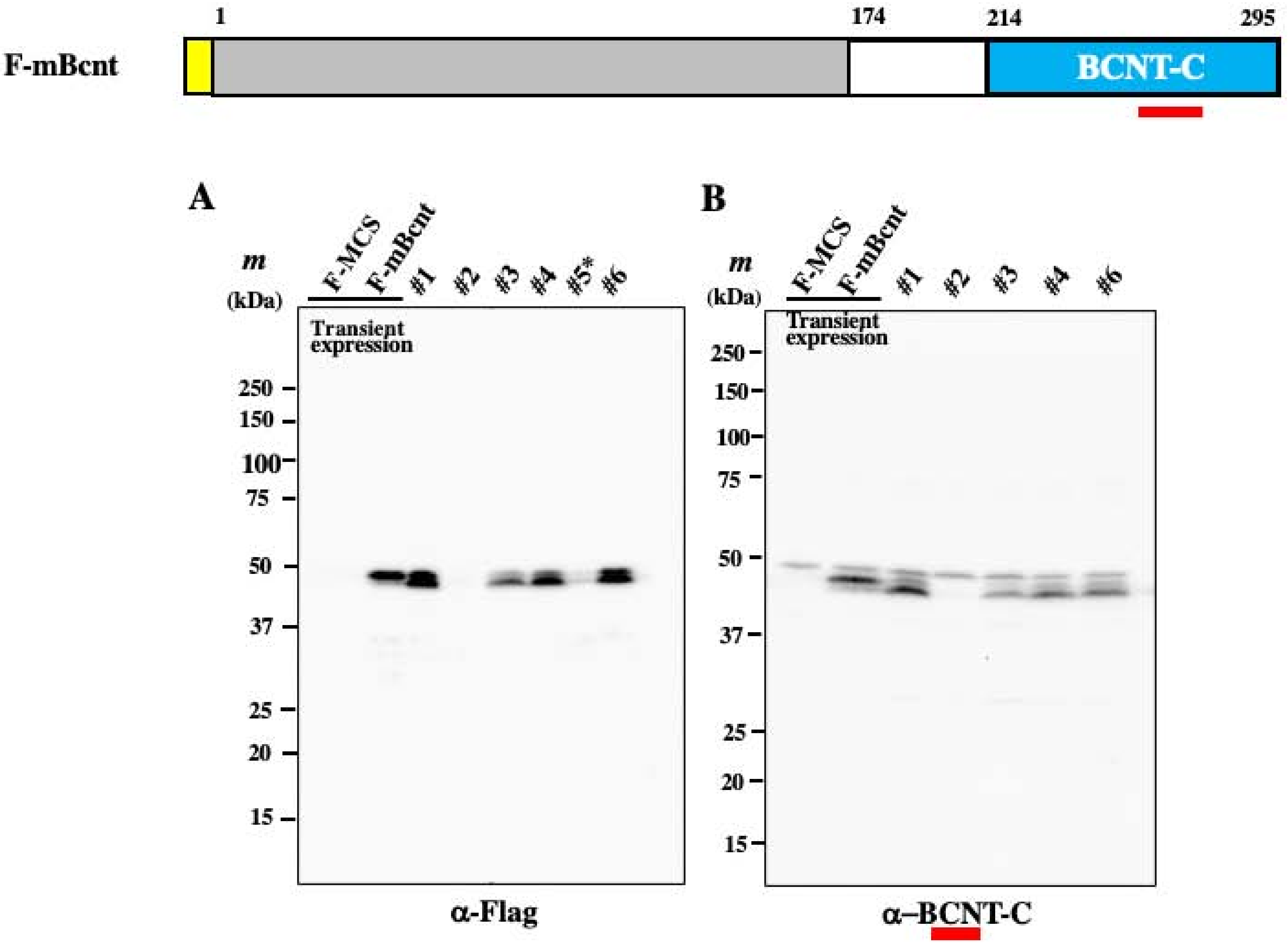

Figure S1. Screening of F-mBcnt expressing T-REx cell colonies by Western blot.

Top panel: Molecular architecture of Flag-tagged mouse Bcnt/Cfdp1. The protein shown in a large outline comprises the acidic N-terminal region, Lys/Glu/Pro-rich 40 amino acids(white box), a highly conserved C-terminal region (BCNT-C domain, blue box), and Flag-tag at the N-terminus (yellow box). The numbers above the outline show the amino acid residues of mBcnt/Cfdp1. The underlined red bar presents the location of the immunogen for generation of anti-BCNT-C Ab. Cell extracts of several G418 resistant colonies from F-mBcnttransfectants(#1~6), and of MCS- or F-mBcnt transiently expressed cultures (all equivalent to ~ 5 x 10^4 cells per lane, shown at the top of each lane), were subjected to Western blot analysis using anti-Flag Tag Ab (A) or anti-BCNT-C Ab (B). #5* indicates a loss of the sample during applicationto the gel (A).

Sup Fig S2

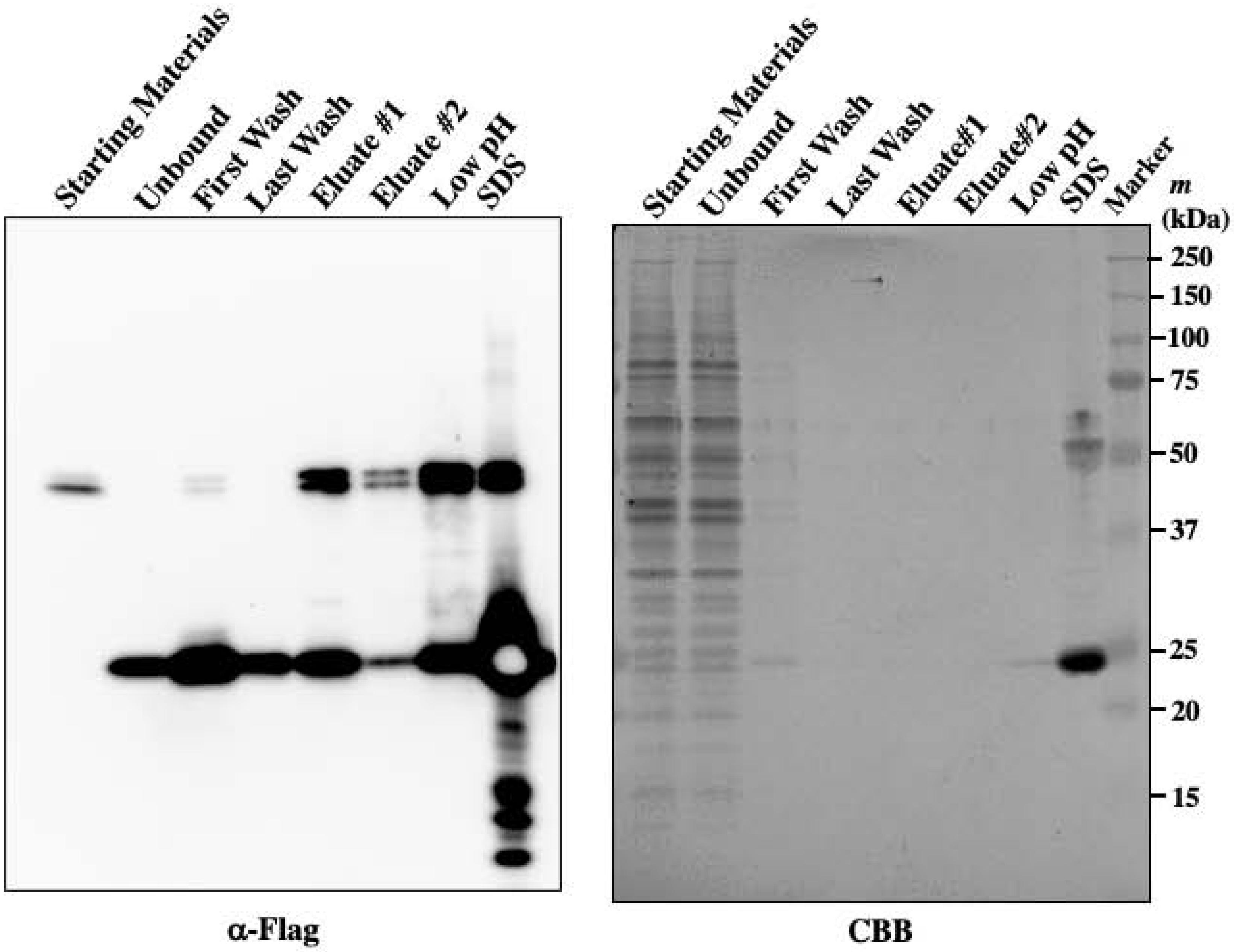
Starting Materials
Unbound
First Wash
Last Wash
Eluate #1
Eluate #2
Low pH
SDS
α-Flag
Starting Materials
Unbound
First Wash
Last Wash
Eluate#1
Eluate#2
Low pH
SDS
Marker
m (kDa)
250
150
100
75
50
37
25
20
15
CBB

Figure S2. Isolation of F-mBcnt from T-RExcolony #1 usinganti-Flag Ab-conjugatedagarose beads.

The supernatant of the #1 colony extract was mixed with anti-Fla-tag antibody-conjugatedagarose beads and incubated.The bound fraction was washed, eluted with Flag (DYKDDDDK) peptide sequentially (Eluate#1 and #2), and followed by glycine-HCl, pH 2.5 buffer (Low pH). Finally, the agarose was boiled in SDS/PAGE sample buffer and its supernatant was obtained (SDS). All of the samples were subjected to Western blot analysis with anti-Flag Tag Ab followed by HRP-conjugated anti-mouse IgG light chain specific Ab. Finally, the filter was stained with Coomassie Brilliant Blue (CBB, right panel).

Sup Fig S3

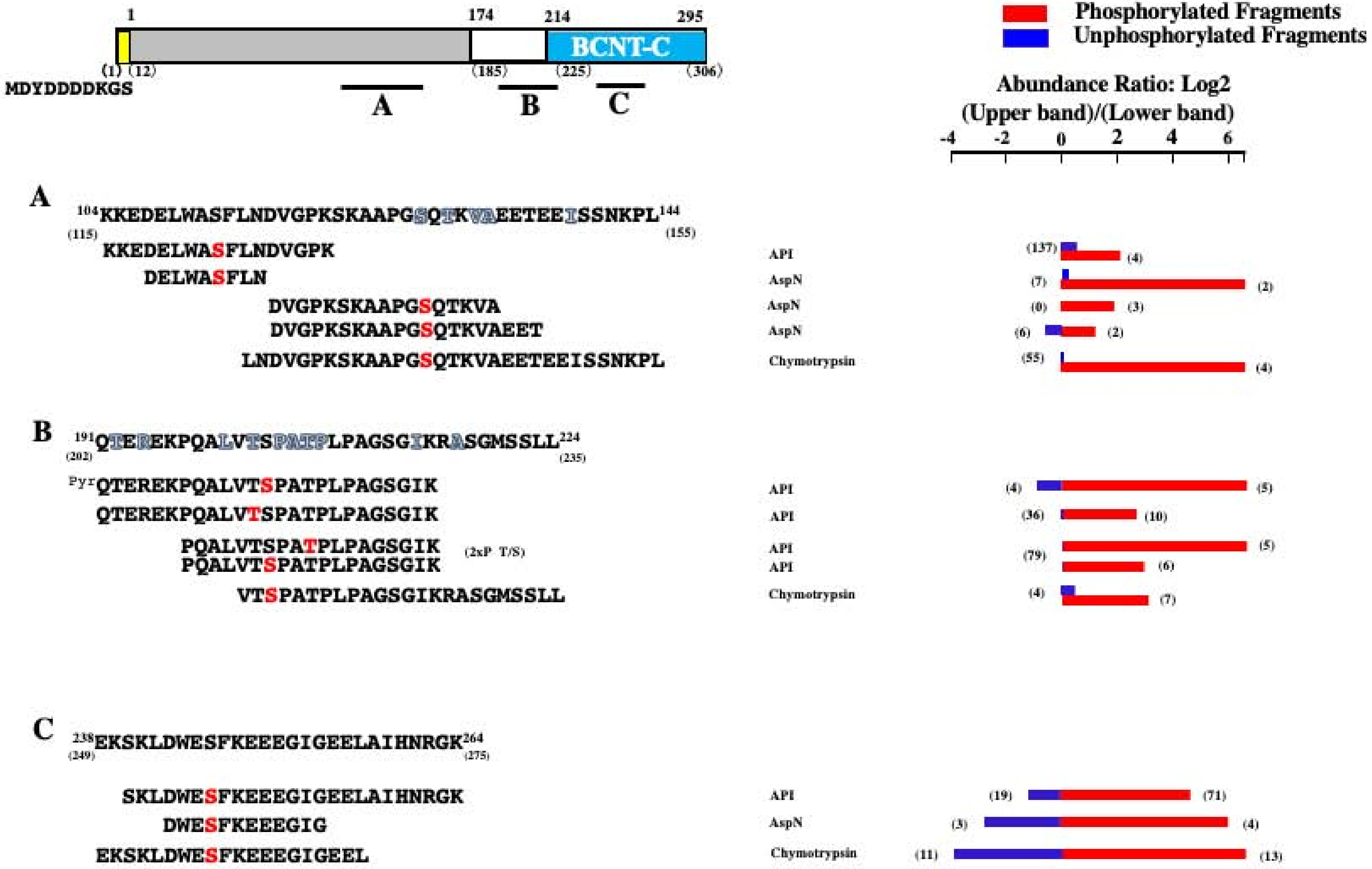
1
174
214
295
BCNT-C
(1) (12)
(185)
(225)
(306)
MDYDDDDKGS
A
B
C
Phosphorylated Fragments
Unphosphorylated Fragments
Abundance Ratio: Log2
(Upper band)/(Lower band)
-4 -2 0 2 4 6
A
104KKEDELWASFLNDVGPKSKAAPGSQTKVAEETEEISSNKPL144
(115)
(155)
KKEDELWASFLNDVGPK
DELWASFLN
DVGPKSKAAPGSQTKVA
DVGPKSKAAPGSQTKVAEET
LNDVGPKSKAAPGSQTKVAEETEEISSNKPL
API
AspN
AspN
AspN
Chymotrypsin
(137) (4)
(7) (2)
(0) (3)
(6) (2)
(55) (4)
B
191QTEREKPQALVTSPATPLPAGSGIKRASGMSSLL224
(202)
(235)
PyrQTEREKPQALVTSPATPLPAGSGIK
QTEREKPQALVTSPATPLPAGSGIK
PQALVTSPATPLPAGSGIK (2xP T/S)
PQALVTSPATPLPAGSGIK
VTSPATPLPAGSGIKRASGMSSLL
API
API
API
API
Chymotrypsin
(4) (5)
(36) (10)
(79) (5)
(6)
(4) (7)
C
238EKSKLDWESFKEEEGIGEELAIHNRGK264
(249)
(275)
SKLDWESFKEEEGIGEELAIHNRGK
DWESFKEEEGIG
EKSKLDWESFKEEEGIGEEL
API
AspN
Chymotrypsin
(19) (71)
(3) (4)
(11) (13)

Figure S3. Differential phosphorylation between the upper and lower bands of F-mBcnt.

Top panel: Molecular architecture of Flag-tagged mouse Bcnt/Cfdp1 as shown in Supplementary figure S1. The numbers above and below the F-mBcnt structure show its amino acid residues withoutand with Flag tag, respectively. Three black bars A, B, and C indicate the grouping regions of focused phosphorylation sites. Each upper and lower band of F-mBcnt from Fig. 1B was digested with three proteases (API, AspN, and Chymotrypsin) and subjected to LC-MS/MS analysis. All of the identified peptides are listed in Supplementary Table S1, and their typical phosphorylated fragments and their unphosphorylated counterparts are represented in this figure. Amino acid sequence shown in each top line of A, B, and C, represents each focused region, and their numbers at the N-terminus and the C-terminus correspond to the amino acid residue of mBcnt, respectively. **X**s are different amino acid residues from human BCNT/CFDP1 andred letters show the identified phosphorylated sites. In the right side of the panel, red bars indicate the ratios of the amounts of identified phosphorylated fragments in the upper band compared to those in the lower band, and blue bars show the corresponding ratios of their unphosphorylated fragments, respectively. The numbers of peptide-spectrummatch (PSM)values are presented in parentheses.

Sup Fig S4

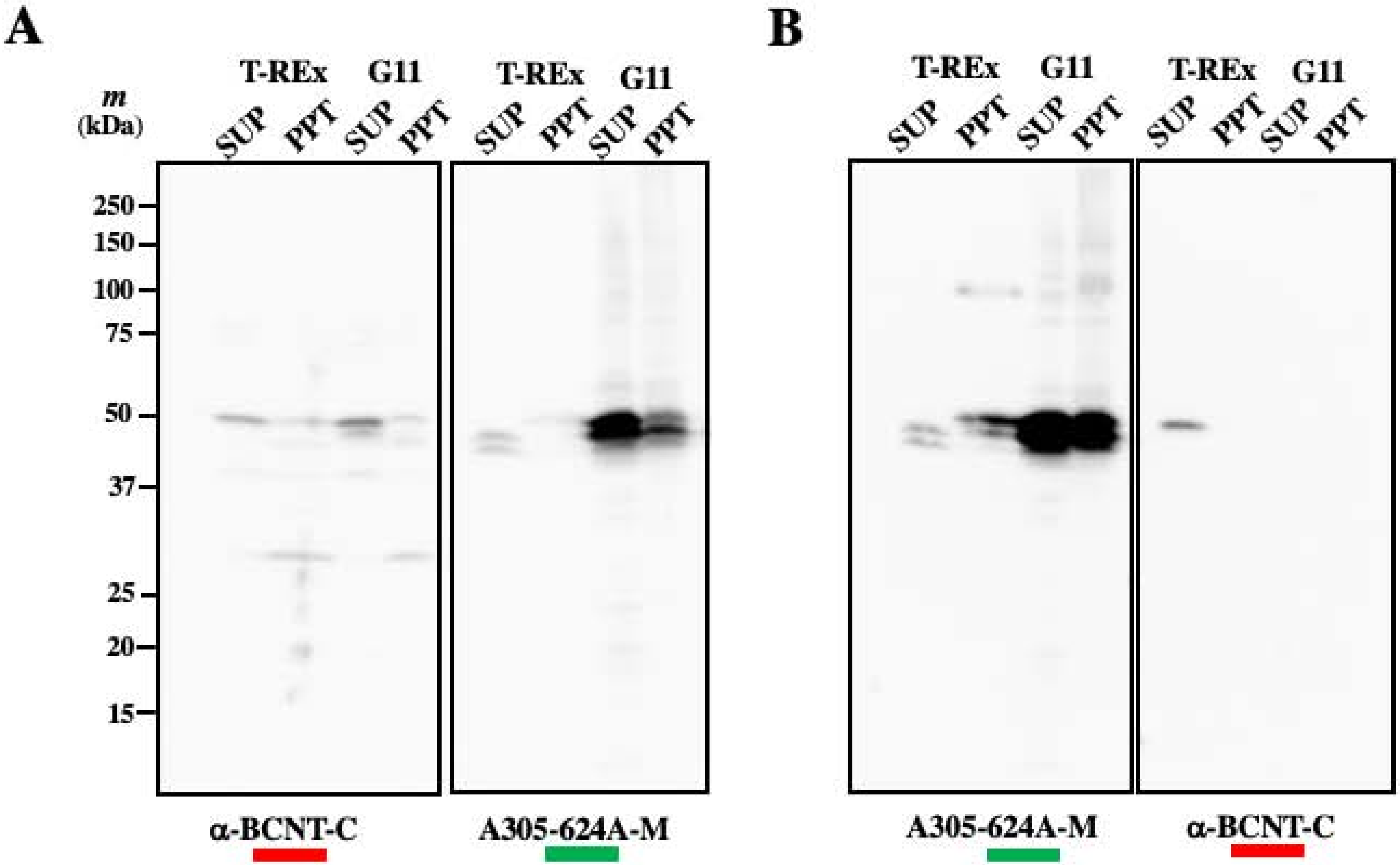
A
B
T-REx
G11
T-REx
G11
m
(kDa)
SUP
PPT
SUP
PPT
SUP
PPT
SUP
PPT
250
150
100
75
50
37
25
20
15
α-BCNT-C
A305-624A-M
T-REx
G11
T-REx
G11
SUP
PPT
SUP
PPT
SUP
PPT
SUP
PPT
A305-624A-M
α-BCNT-C

Figure S4. Comparative assessment of western blot signals between the anti-BCNT-C antibody and A305-624A-M.

The supernatants (SUP) and their pellets (PPT) of T-REx or G11 cells (His-tagged human BCNT/CFDP1 constitutively expressing clone) were prepared from each cell lysate by centrifugation at 25000 x *g*. Equal amounts of protein (20 μg) were subjected to a western blot analysis with either anti-BCNT-C Ab or A305-624A-M **(A)**. After obtaining their images, each filter was stripped and reprobed with the exchanged Abs **(B)**.

Sup Fig S5

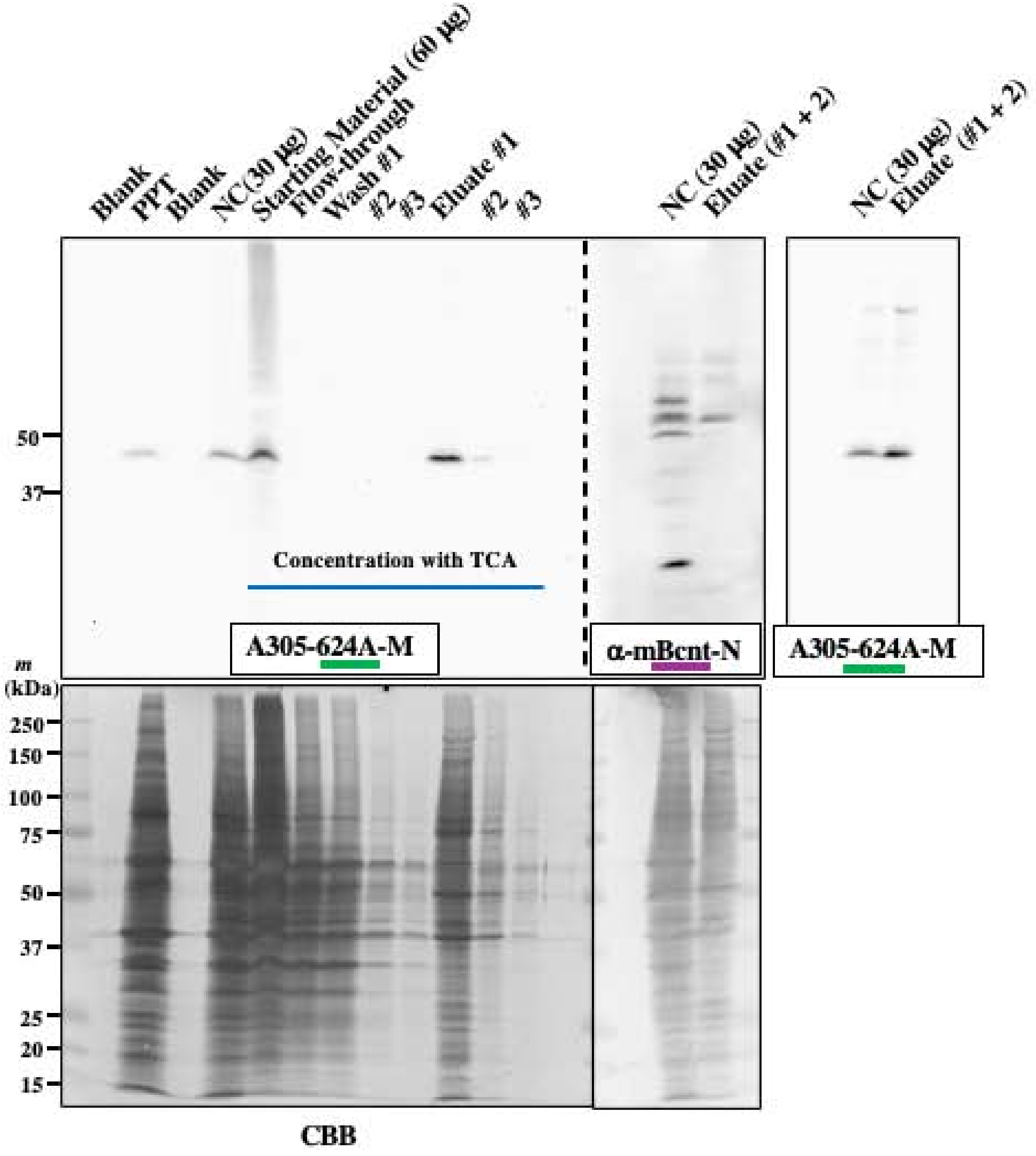

Blank
PPT
Blank
NC(30 μg)
Starting Material (60 μg)
Flow-through
Wash #1
#2
#3
Eluate #1
#2
#3
NC (30 μg)
Eluate (#1 + 2)
NC (30 μg)
Eluate (#1 + 2)
50
37
Concentration with TCA
A305-624A-M
α-mBcnt-N
A305-624A-M
m
(kDa)
250
150
100
75
50
37
25
20
15
CBB

Figure S5. Enrichment of bovine Bcnt/Cfdp1 with Phos-tag agarose.

The supernatant of the bovine placenta extract in RIPA buffer (Starting material, SM) was mixed with Phos-tag agarose and incubated. After washing three times (Wash #1, 2, 3), the bound portion was sequentially eluted (Eluate #1, 2, 3), and each sample was concentrated with TCA. On the other hand, the pellet fraction, which contained ~9-fold concentrated protein compared to the supernatant, was prepared (PPT). All of the samples were boiled in SDS/PAGE sample buffer and then subjected to a western blot analysis with A305-624-M (top, left filter). The samples before and after Phos-tag treatment, corresponding to Non-concentated (NC) and the pooled Eluates (#1 / 2), respectively, were also analyzed with anti-mBcnt-N Ab (top, middle filter) followed by reprobing with A305-624-M (top, right filter). Finally, the filters were stained with Coomassie Brilliant Blue (CBB, bottom panel).

Sup Fig S6

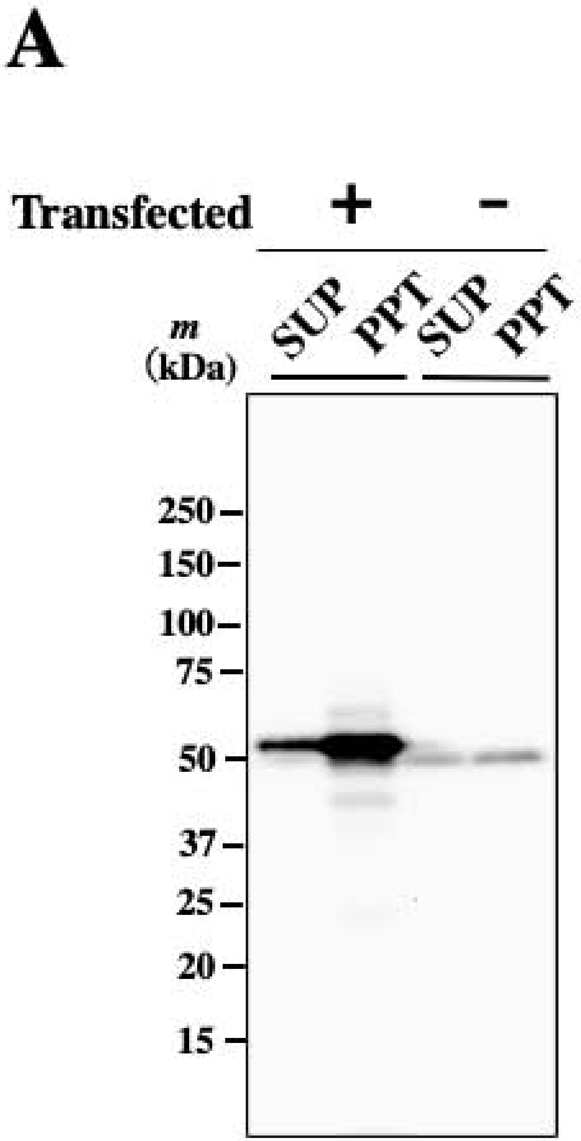
A
Transfected
+
-
SUP
PPT
SUP
PPT
m
(kDa)
250
150
100
75
50
37
25
20
15

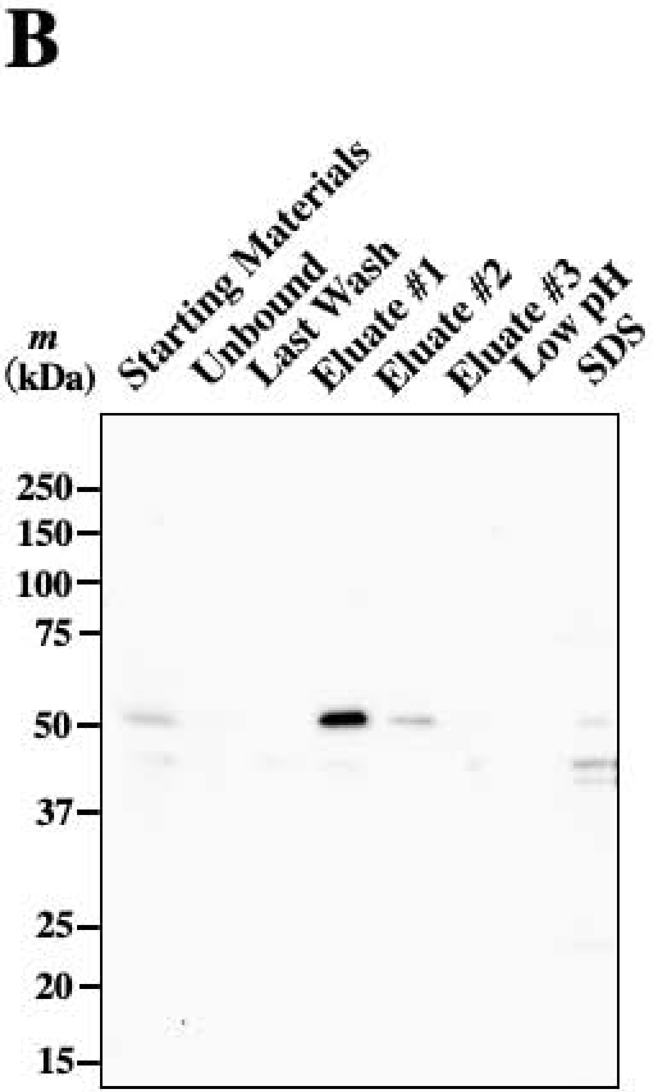
B
Starting Materials
Unbound
Last Wash
Eluate #1
Eluate #2
Eluate #3
Low pH
SDS
m
(kDa)
250
150
100
75
50
37
25
20
15

Figure S6. Isolation of Nono (p54rnb) usinganti-HisAb-conjugatedagarose.

(A) Evaluation of anti-p54nrb Ab. T-REx 293 Clone G11 cells were transfected with Hemagglutinin-tagged P54nrb pcDNA3.1 (a gift from Dr. Atsushi Yokoyama, Tohoku University, Sendai) and the cell lysateswere prepared after culturing for total 70 h, and centrifuged at 25000 x *g*, 30 min. The protein ratio between the supernatant (SUP) and the particulate (PPT) was 8:1. Their constant amounts of protein (18 μg/lane) were subjected to a Western blot analysis with anti-p54nrb antibody (250 ng/mL,GeneTex, GTX101419). **(B)** Isolation of Nonowith anti-His tag antibody-conjugated agarose beads. The supernatant of T-REx cell lysate was applied to anti-His-tag antibody-conjugated agarose beads, and after washing, the bound proteins to agarosewere eluted with His-tag peptide as previously described [16], followed by with glycine-HCl buffer (pH 2.5), and finally boiled in SDS/PAGE sample buffer as shown in Supplementary figure S2. All of the samples were treated in SDS/PAGE sample buffer and subjected to a western blot analysis as described in (**A**).
