# Supplementary material for: Overcoming off-targets: assessing Western blot signals for Bcnt/Cfdp1, a tentative component of the chromatin remodeling complex
Source: Biosci Rep. 2020 Jun 9;40(6):BSR20194012. doi: 10.1042/BSR20194012 (PMC7284322; doi:10.1042/BSR20194012)
Supplement: Supplementary Tables S1-S4 [file BSR-2019-4012_supp1.zip › BSR-2019-4012_supp-caption.pdf]

Table S1: Protease-digested fragments of the upper and lower bands of F-mBcnt.

Table S2: Whole expression profile in Cfdp-1 (Mutant) and vdR2-4 (Wild type) cells.

Table S3: Differential expression profiles of *Bcnt/Cfdp1* flanking genes and internal control genes between Cfdp1-K1 and vdR2-4 cells.

Table S4 : List of reagents and materials.
